# Ras/Raf/MEK/ERK and PI3K/PTEN/Akt/mTOR Inhibitors: Rationale and Importance to Inhibiting These Pathways in Human Health

**DOI:** 10.18632/oncotarget.240

**Published:** 2011-03-11

**Authors:** William H. Chappell, Linda S. Steelman, Jacquelyn M. Long, Ruth C. Kempf, Stephen L. Abrams, Richard A. Franklin, Jörg Bäsecke, Franca Stivala, Marco Donia, Paolo Fagone, Graziella Malaponte, Maria C. Mazzarino, Ferdinando Nicoletti, Massimo Libra, Danijela Maksimovic-Ivanic, Sanja Mijatovic, Giuseppe Montalto, Melchiorre Cervello, Piotr Laidler, Michele Milella, Agostino Tafuri, Antonio Bonati, Camilla Evangelisti, Lucio Cocco, Alberto M. Martelli, James A. McCubrey

**Affiliations:** ^1^ Department of Microbiology and Immunology, Brody School of Medicine at East Carolina University; ^2^ Department of Physics, Greenville, NC 27858 USA; ^3^ Department of Medicine University of Göttingen, Göttingen, Germany; ^4^ Department of Biomedical Sciences, University of Catania, Catania, Italy; ^5^ Department of Immunology, Institute for Biological Research “Sinisa Stankovic”, University of Belgrade, Belgrade, Serbia; ^6^ Department of Internal Medicine and Specialties, University of Palermo, Palermo, Italy; ^7^ Consiglio Nazionale delle Ricerche, Istituto di Biomedicina e Immunologia Molecolare “Alberto Monroy”, Palermo, Italy; ^8^ Department of Medical Biochemistry Jagiellonian University Medical College, Krakow, Poland; ^9^ Regina Elena Cancer Center, Via Elio Chianesi n.53, Rome 00144, Italy; ^10^ University of Rome, La Sapienza, Department of Hematology-Oncology, Via Benevento 6, Rome 99161, Italy; ^11^ University Hospital of Parma, Unit of Hematology and Bone-Marrow Transplantation, Via Gramsi n.14, Parma 43100, Italy; ^12^ Dipartimento di Scienze Anatomiche Umane e Fisiopatologia dell'Apparato Locomotore, Università di Bologna, Bologna, Italy; ^13^ IGM-CNR, Sezione di Bologna, C/o IOR, Bologna, Italy

**Keywords:** Targeted Therapy, Combination Therapy, Drug Resistance, Cancer Stem Cells, Aging, Senescence, Raf, Akt, PI3K, mTOR

## Abstract

The Ras/Raf/MEK/ERK and PI3K/PTEN/Akt/mTOR cascades are often activated by genetic alterations in upstream signaling molecules such as receptor tyrosine kinases (RTK). Integral components of these pathways, Ras, B-Raf, PI3K, and PTEN are also activated/inactivated by mutations. These pathways have profound effects on proliferative, apoptotic and differentiation pathways. Dysregulation of these pathways can contribute to chemotherapeutic drug resistance, proliferation of cancer initiating cells (CICs) and premature aging. This review will evaluate more recently described potential uses of MEK, PI3K, Akt and mTOR inhibitors in the proliferation of malignant cells, suppression of CICs, cellular senescence and prevention of aging. Ras/Raf/MEK/ERK and Ras/PI3K/PTEN/Akt/mTOR pathways play key roles in the regulation of normal and malignant cell growth. Inhibitors targeting these pathways have many potential uses from suppression of cancer, proliferative diseases as well as aging.

## INTRODUCTION

The Ras/Raf/MEK/ERK and Ras/PI3K/PTEN/Akt/mTOR signaling cascades have been extensively studied over the past few decades. In this time there have been breakthroughs in the discovery of pathway components, the mechanisms by which they relay their signals and how mutations of these components can lead to aberrant signaling and uncontrolled proliferative diseases. Research has also lead to the development of inhibitors that specifically target critical elements of these pathways in anticipation of ameliorating patient survival. This review will discuss some of the current inhibitors, their targets and how they are being used to treat cancer and other proliferative diseases including aging.

**Figure 1:**
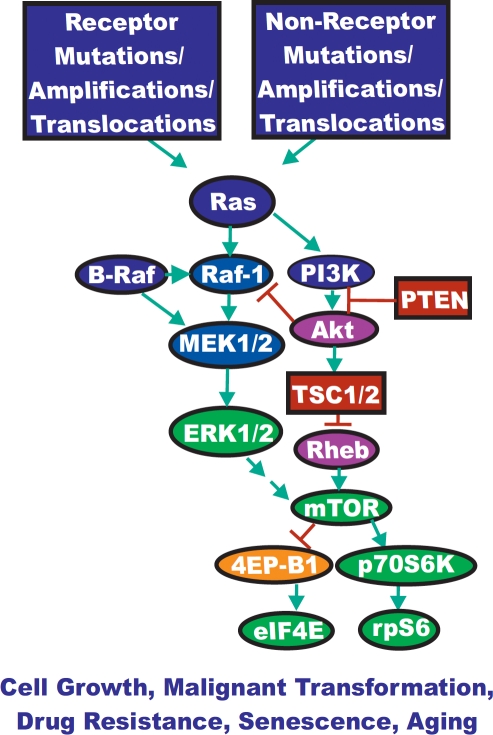
Dysregulated Expression of Upstream Receptors and Kinases Can Result in Activation of the Ras/Raf/MEK/ERK and Ras/PI3K/PTEN/Akt/mTOR Pathway Sometimes dysregulated expression of growth factor receptors occurs by increased expression, genetic translocations or genomic amplifications which can lead to activation of the Ras/Raf/MEK/ERK and Ras/PI3K/PTEN/Akt/mTOR pathways. Alternatively chromosomal translocations can occur in non-receptor kinases and other genes which result in activation of these pathways. Genes in the Ras/Raf/MEK/ERK and Ras/PI3K/PTEN/Akt/mTOR pathways that have activating mutations detected in human cancer and proliferative diseases are indicated in blue ovals. Genes overexpressed in certain cancers are indicated in purple ovals. Tumor suppressor genes mutated in human cancer are indicated in red rectangles. Other key genes are indicated in green ovals. Genes inactivated by the Ras/PI3K/PTEN/Akt/mTOR pathway are indicated in orange ovals. Green arrows indicate activating events in pathways. Blocked red arrows indicating inactivating events in pathway.

Signaling through the Ras/Raf/MEK/ERK and Ras/PI3K/PTEN/Akt/mTOR pathways are carefully orchestrated events generally starting from the cell surface and leading to controlled gene expression within the nucleus. Regulation of these pathways is mediated by a series of kinases, phosphatases and various exchange proteins. Mutations occur in many of these pathway elements leading to uncontrolled regulation and aberrant signaling. An overview of the effects of mutations and the activation of these signaling pathways is presented in Figure 1. Deregulated signaling can lead to unrestrained cellular growth and proliferation ultimately resulting in tumor formation or abnormal cellular growth and premature aging. As such, a great deal of research has been aimed to target these mutated proteins to prevent abnormal signaling [1-5].

## MUTATIONS OR ALTERED EXPRESSION OF THESE PATHWAYS CAN LEAD TO SENSITIVITY TO THERAPY

**Figure 2:**
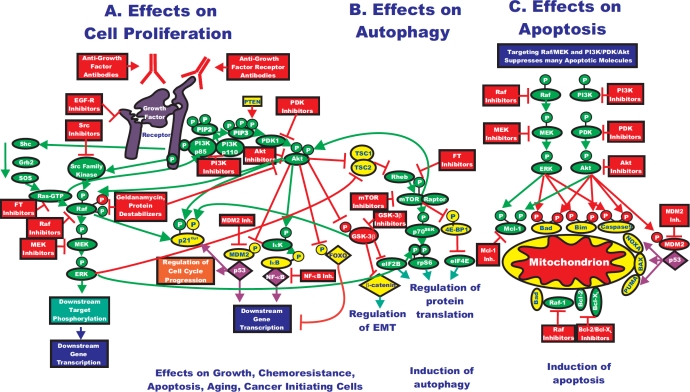
Rationale for Targeting Both the Ras/Raf/MEK/ERK and Ras/PI3K/PTEN/Akt/mTOR Pathways for Suppressing Cancer Growth A: The Ras/Raf/MEK/ERK and Ras/PI3K/PTEN/Akt/mTOR pathways are both activated by upstream receptor ligation and frequently co-regulate many downstream targets in parallel. Thus for effective elimination of many cancers or prevention of aging, it may be necessary to target both signaling pathways. Activation of these pathways could also result in increased transcription of many genes that promote cellular growth and malignant transformation. B. Inhibition of mTOR can result in the induction of autophagy, which is a very important mechanism of cell death, especially in solid tumors. C. As described previously, both the Ras/Raf/MEK/ERK and Ras/PI3K/PTEN/Akt/mTOR pathways regulate the activity of apoptotic proteins by post-translational mechanisms. Targeting this pathway may also contribute to the induction of apoptosis. Signaling molecules promoting phosphorylation events are indicated in green. Stimulatory signaling events are indicted in green lines with a green arrow before the target of the phophorylation. Small molecule inhibitors are indicated in red. Inhibitory phosphorylation events are indicated in red lines with a block on the end before the target of the inhibition. Inhibitory signaling or proapoptotic molecules or inactivated molecules are indicated in yellow. A growth factor and a growth factor receptor are indicated in purple. Active transcription factors are indicated in purple diamonds. Inactivated transcription factors are indicated in yellow diamonds.

Some cancer cells carrying BRAF mutations are highly sensitive to MEK inhibitors, while cells lacking these BRAF mutations or containing RAS or epidermal growth factor receptor (EGFR) mutations are resistant [5, 6]. Increased Akt activity may actually render cells and patients sensitive to Akt as well as downstream mTOR inhibitors. The formation of the rapamycin-sensitive mTORC1 complex (consisting of mTOR, regulatory“associated protein of mTOR [Raptor], DEPTOR and mLST8) in certain cancer cells that overexpress activated Akt may be altered in comparison to cells that do not overexpress Akt. In cells that express activated Akt, Akt may phosphorylate TSC-2 resulting in its inactivation. The mTORC1 complex is formed and downstream p70^S6K^ and 4E-BP1 are phosphorylated, allowing the dissociation of eIF-4E, ribosome biogenesis and protein synthesis. In contrast, in the absence of Akt activation, this complex should not be formed. Rapamycin targets this complex; hence the cells that express elevated levels of activated Akt cells may be more sensitive to rapamycin than the cancer cells that do not express high levels of activated Akt. In the cells that do not express elevated levels of activated Akt, this complex should be transiently assembled after growth factor treatment. In contrast, the assembly of the rapamycin-insensitive mTORC2 complex (consisting of rapamycin insensitive companion of mTOR [Rictor], mTOR, DEPTOR, mLST8) should be lower in the cells that express elevated levels activated Akt than in those cells that do not as there is equilibrium between the mTORC1 and mTORC2 complexes. The significance of these complex biochemical signaling events is that cancer cells that overexpress activated Akt or lack PTEN expression have an Achilles heel with regards to therapeutic intervention as they are highly sensitive to rapamycin treatment. An overview of the interactions between the Ras/Raf/MEK/ERK and PI3K/PTEN/Akt/mTOR pathways and the effects of these pathways on growth, autophagy and apoptosis is presented in Figure 2.

## OVERVIEW OF PATHWAY INHIBITORS

Effective inhibitors specific for many of the key components of the Ras/Raf/MEK/ERK and Ras/PI3K/PTEN/mTOR pathways have been developed [7-35]. In many cases, these inhibitors have been examined in clinical trials. Furthermore, inhibitors that target the mutant but not the wild type (WT) alleles of various genes (*e.g*., *BRAF* and *PIK3CA*) either have been or are being characterized. Thus specific inhibitors have been made and some are currently in the clinic. Targeting some components of these pathways has proven clinically effective and in some of the diseases have a very large market with few effective treatments [(*e.g*., Sorafenib and hepatocellular carcinoma (HCC)] [7].

## RAF/MEK INHIBITORS

Raf inhibitors have been developed and some are being used for therapy while others are being evaluated in clinical trials (See Table 1). Some inhibitors (*i.e,* Sorafenib, Bayer) were initially thought to specifically inhibit Raf but have been subsequently shown to have multiple targets (*e.g*., VEGF-R, Flt-3, PDGF-R). However, that does not preclude their usefulness in cancer therapy. Sorafenib is approved for the treatment of certain cancers (*e.g.,* renal cell carcinoma (RCC) and patients with unresectable HCC and is currently being further evaluated in the Sorafenib Hepatocellular carcinoma Assessment Randomized Protocol (SHARP) trial, which demonstrated that the drug was effective in prolonging median survival and time-to-progression in patients with advanced HCC. Sorafenib is generally well tolerated in HCC patients with a manageable adverse events profile [7]. MEK inhibitors have also been examined for treating HCC in mouse models [8,9] but they do not appear to be as effective as Sorafenib, most likely due to the broad specificity of Sorafenib, which inhibits other targets besides Raf.

**Table 1:** Inhibitors of Raf/MEK and PI3K/PDK/Akt/mTOR

Inhibitor	Target(s)	Cancer Examined	Clinical Trials	Company	Ref.
*Ras Inhibitors*					
**Tipifarnib (Zarnestra™, R115777)**	Ras, farnesyl-transferase, Rheb	AML, lymphoma, breast, glioma, melanoma	Phase I, II, III	Johnson & Johnson	121, 122, www.clinicaltrials.gov
*Raf Inhibitors*					
**BAY 43-9006 (Nexavar®, Sorafenib tosylate)**	Raf, VEGFR2, VEGFR3, PDGF-R, c-Kit, c-Fms, Flt-3	renal cell carcinoma, HCC, melanoma, leukemias	Phase I, II, III	Bayer	7, 31, 33, 58, 59, 61, 71, 73, 79, 158, www.clinicaltrials.gov
**AAL-881**	Raf	thyroid, glioma	Preclinical	Novartis	96, 97
**LBT-613**	Raf	glioma, thyroid	Preclinical	Novartis	96, 157
**RAF265**	B-Raf, Raf-1 (c-Raf), A-Raf, B-Raf^V600E^, VEGFR-2	melanoma	Phase I	Novartis	Data on file. Novartis Pharma AG, Basel, Switzerland (Internet)
**XL281**	B-Raf, c-Raf, B-Raf^V600E^	colorectal, papillary thyroid, ovarian, prostate, carcinoid tumors, melanoma	Phase I	Exelixis/Bristol Myers Squibb	98
**SB-590885**	Raf, B-Raf^V600E^	melanoma	Preclinical	GlaxoSmithKline	99, 155
**PLX-4720**	Raf, B-Raf^V600E^	melanoma	Preclinical	Plexxikon/Roche	10
**PLX-4032**	Raf, B-Raf^V600E^	melanoma, thyroid, ovarian, solid tumors	Phase I	Plexxikon/Roche	11, www.clinicaltrials.gov
**L-779,450**	Raf	leukemia	Preclinical	Merck	13, 106
**GW5074**	Raf-1 (c-Raf)	melanoma, glioblastoma	Preclinical	GlaxoSmithKline	105, 156
**SB-699393**	Raf		Preclinical	GlaxoSmithKline	106
*MEK inhibitors*					
**CI-1040 (PD-184352)**	MEK1, MKK5	colorectal, NSCLC, pancreatic, kidney, melanoma, breast	Phase I, II (discontinued)	Pfizer	13, 17, 25, 27, 29, 74, 77 126 www.clinicaltrials.gov
**PD0325901**	MEK1/2	breast, colon, NSCLC, melanoma	Phase I, II (discontinued)	Pfizer	5, 13, 28, 29, 34, 35, 39, 74, 191 www.clinicaltrials.gov
**XL518**	MEK		Phase I	Exelixis	145, Exelixis (internet), www.clinicaltrials.gov
**Selumetinib (AZD6244, ARRY-142886)**	MEK	melanoma, HCC, pancreatic, colon, lung, breast	Phase I, II	Astra Zeneca/Array BioPharma	3, 8,13, 21, 23, 24, 61, 62, 78, 84 www.clinicaltrials.gov
**RDEA119 (BAY 869766)**	MAP2K1 (MAPK/ERK kinase 1)	advanced tumors	Phase I, II	Ardea/Bayer	15, www.clinicaltrials.gov
**PD098059**	MEK1/2	advanced hematological and advanced solid cancers	Preclinical	Parke-Davis/Pfizer	14, 81, 102, 191, 215, 251, 256
**U0126**	MEK1/2	advanced hematological and advanced solid cancers	Preclinical	DuPont Pharmaceuticals	14, 81, 83, 104, 215, 256, 267
**SL-327**	MEK1/2	not evaluated for use in cancer treatment	Preclinical	DuPont Pharmaceuticals	159
*PI3K/Akt/mTOR inhibitors*					
**UCN-01**	PDK-1, Chk1, PKC isoforms	leukemia, lymphoma, ovarian, peritoneal cavity, fallopian tube	Phase I, II	Kyowa Hakko Kogyo Co., Ltd./Keryx Biopharmaceuticals	108, www.clinicaltrials.gov
**(NVP)-BAG956**	PDK, p110 PI3Ks (except for β isoforms)	leukemia, melanoma	Preclinical	Novartis	111, 112, 153
**Celecoxib (Celebrex®)**	PDK-1, COX-2	lung, prostate, H&N,	Phase I, II	Pfizer	36, 162, 163, www.clinicaltrials.gov
**OSU-03012**	PDK-1	prostate, glioma, leukemia, HCC, breast	Preclinical	Arno Therapeutics/The Ohio State University	131-133
**BX-795**	PDK-1, ERK8, TBK1, IKK-ε	breast, prostate, colon, melanoma, pancreatic, cervical	Preclinical	Berlex/Bayer	124-126
**BX-912**	PDK-1	breast, prostate, colon, melanoma, pancreatic, cervical	Preclinical	Berlex/Bayer	124-126
**BX-320**	PDK-1	breast, prostate, colon, melanoma, pancreatic, cervical	Preclinical	Berlex/Bayer	124-126
**AR-12**	PDK-1, PI3K, Akt	breast, colon, lung, prostate, lymphoma	Phase I	Arno Therapeutics	Arno Therapeutics (internet), www.clinicaltrials.gov
**KP372-1**	PDK-1, Akt, Flt3	AML, thyroid, glioblastoma	Preclinical	Kinetek Pharmaceuticals	47, 140, 141
**LY294002**	PI3K, other related kinases	advanced hematological and advanced solid cancers	Preclinical	Lilly	107, 126, 139, 215, 251, 256, 267
**PWT-458**	PI3K	NSCLC, glioblastoma, renal	Preclinical	Wyeth/Pfizer	136, 137
**PX-866**	PI3K	glioma, breast, colon, prostate, NSCLC, pancreatic advance solid tumors	Phase I	Oncothyreon Inc.	134, 135, www.clinicaltrials.gov
**CAL-101**	PI3K (p110δ)	leukemias, lymphomas, myeloma	Phase I	Calistoga Pharmaceuticals	Calistoga Pharmaceuticals (Internet), www.clinicaltrials.gov
**XL-147**	PI3Ks	NSCLC, solid tumors	Phase I	Exelixis/Sanofi-Aventis	142, Exelixis (internet), www.clinicaltrials.gov
**ZSTK474**	PI3Ks	NSCLC, melanoma, ovarian, prostate,	Preclinical	Zenyaku Kogyo Co. Ltd	143, 144
**GDC-0941**	PI3K (p110α), Flt3	lymphoma, NSCLC, breast, solid tumors	Phase I	PIramed Pharma/Roche/Genetech	146-148, www.clinicaltrials.gov
**(NVP)-BEZ235**	PI3K, mTOR	breast, glioma, melanoma, pancreatic	Phase I, II	Novartis	54, 149, 150, 170 www.clinicaltrials.gov
**AS-252424**	PI3Ks (p110γ)		Preclinical	Merck Serona	154
**TGX-221**	PI3K (p110β)	treatment for coronary heart disease, not evaluated for use in cancer treatment	Preclinical	Alexis/Enzo Life Sciences, Inc.	160, 161
**XL-765**	PI3K, mTOR	glioma, NSCLC	Phase I	Exelixis/Sanofi-Aventis	145, Exelixis (internet), www.clinicaltrials.gov
**Wortmannin**	PI3K, mTOR, DNA-PK, MAPK	advanced hematological and advanced solid cancers	Preclinical		126, 127, 138, 139
**PI-103**	p110 PI3Ks, mTORC1/2, DNA-PK	glioma, prostate, colon, NSCLC	Preclinical	PIramed Pharma/Roche	44, 126-130
**Perifosine (KRX-0401)**	Akt, MEK 1/2, ERK 1/2, JNK	multiple myeloma, leukemias, NSCLC, advance solid tumors	Phase I, II	AEterna Zentaris Inc./Keryx Biopharmaceuticals	48, 109, 110, 172, 174, 175 www.clinicaltrials.gov (internet)
**Triciribine (API-2)**	Akt 1, 2, 3	AML, advanced hematological cancer	Phase I	VioQuest Pharmaceuticals	45, 55, 166 www.clinicaltrials.gov
**SR13668**	Akt	breast, prostate, ovarian	Preclinical	SRI International	123, SRI International (internet)
**AR-67 (DB-67)**	Akt	advanced solid tumors	Phase I, II	Arno Therapeutics	Arno Therapeutics (internet)
**AR-42**	Akt		Preclinical	Arno Therapeutics	Arno Therapeutics (internet)
**GSK690693**	Akt1, 2, 3	leukemia, lymphoma	Phase I	GlaxoSmithKline	113, 114, www.clinicaltrials.gov
**KP372-1**	Akt, PDK-1, Flt3	leukemia, thyroid, H&N, glioma	Preclinical	QLT Inc.	47, 140
**VQD-002 (API-2)**	Akt	NSCLC, leukemias, lymphomas, prostate	Phase I, II	VioQuest Pharmaceuticals	45, 165
**A-443654**	Akt	hematological and solid cancers	Preclinical	Abbott Laboratories	46, 164
**MK-2206**	Akt	solid tumors	Phase I	Merck	Merck (internet), www.clinicaltrials.gov
**Rapamycin (Sirolimus)**	mTORC1	advanced hematological, advanced solid tumors, HIV, AIDS related malignancies	Phase I, II	Wyeth/Pfizer	2, 50, 51, 53, 63-69, 71, 73, 74, 86-88, 151, 152, 173-185, 191, 212, 215, 216, 255 www.clinicaltrials.gov
**CCI-779 (Torisel®, Temsirolimus)**	mTORC1	leukemia, lymphoma, NSCLC, prostate, colorectal, renal	Phase I, II	Wyeth/Pfizer	2, 50, 53, 70, 115-118, www.clinicaltrials.gov
**RAD001 (Afinitor®, Everolimus)**	mTORC1, mTORC2	cervical, renal, HCC, leukemia, lymphoma	Phase I, II	Novartis	2, 50, 52, 53, 93, 115-118, 157, www.clinicaltrials.gov
**AP-23573 (Ridaforolimus, Deforolimus)**	mTORC1	advanced hematological cancer, prostate, endometrial	Phase I, II	Ariad/Merck	2, 50, 53, 74119, 120, www.clinicaltrials.gov
*Active Site mTOR Inhibitors*					
**AZD-8055**	mTORC1/mTORC2	advanced solid tumors, lymphomas, HCC	Phase I, II	AstraZenica	171, 172, 174-176 www.clinicaltrials.gov
**OSI-027**	mTORC1/mTORC2	advanced solid tumors, lymphomas	Phase I	OSI Pharmaceuticals	171, 172, 174-176 www.clinicaltrials.gov
**INK-128**	mTORC1/mTORC2	advanced cancers, multiple myeloma, Waldenstrom macroglobulinemia	Phase I	Intellikine	172, 174-176 www.clinicaltrials.gov
**PP-242**	mTORC1/mTORC2		Phase I	UCSF	171, 172, 174-176
AML = acute myeloid leukemia, HCC = hepatocellular carcinoma, NSCLC = non-small cell lung carcinoma, H&N = head and neck cancer					

PLX-4720 (Plexxikon/Roche) (R7204) is a mutant B-Raf specific inhibitor that has been used for preclinical studies [10]. PLX-4032 is a B-Raf inhibitor that is being evaluated in clinical trials. PLX-4720 was designed using a unique screening platform developed by Plexxikon that involved the use of structural and medicinal chemistry techniques [10]. This more selective screening approach has resulted in a series of B-Raf inhibitors based on the structural implications of BRAF mutation and which discriminate between the mutant and WT protein. PLX-4720 is orally available and is highly selective for the mutant B-Raf protein. PLX-4720 is effective against melanomas, as well as colorectal tumors and other cancers, with the BRAF^V600E^ mutation. BRAF^V600E^ has been associated with more aggressive tumors and lower rates of patient survival [10]. The IC50 value for PLX-4720 is approximately 3-fold lower in in vitro kinase assays with mutant versus WT B-Raf proteins and demonstrates an approximately 60-fold lower IC50 value in vivo when cell lines with mutant and WT BRAF genes are compared [10]. The IC50 value for PLX-4720 was compared with Sorafenib in a panel of melanomas, colon carcinomas and NSCLC. The BRAF gene status was known in all of these cell lines. The IC50 value for PXL-4720 was approximately 100-fold lower (range: 17.5 to 280 nM) than Sorafenib in melanomas and colon carcinomas that had the BRAF^V600E^ mutation; however, the IC50 value for PLX-4720 was approximately the same as Sorafenib in colon carcinomas and NSCLC without BRAF mutations, but with RAS mutations [10]. PLX-4720 arrests mutant but not WT B-Raf melanoma cells at the G0/G1 cell-cycle stage and initiates apoptosis in these cells. The additional B-Raf inhibitor (PLX-4032) developed by Plexxicon shows promising effects [11].

## NEED FOR GENETIC SCREENING BEFORE TREATMENT WITH RAF KINASE INHIBITORS

It has recently become apparent that it will be critical to determine the genetic status at both B-Raf and Ras before treatment with B-Raf selective inhibitors [12]. Class I B-Raf inhibitors (active conformation inhibitors) such as (PLX4720 and 885-A, a close analog of SB590885) will inhibit B-Raf mutants, however these ATP-competitive B-Raf inhibitors will not inhibit WT B-Raf or mutant Ras. In fact, these B-Raf inhibitors can activate Raf-1 in these cells in the presence of active Ras. 885-A could induce B-Raf binding to Raf-1. PLX-4720 can, to a lesser extent, induce B-Raf binding to Raf-1 when the ERK-mediated negative feedback loop on B-Raf was inhibited with a MEK inhibitor. These binding events were determined to require the present of activated Ras (WT or mutant), which may be necessary for the translocation from the cytoplasm to the membrane and assembly into the signaling complex. This has therapeutic implications, as in patients with mutant *RAS*, if they are treated with certain B-Raf inhibitors, B-Raf can bind and activate Raf-1 and promote the oncogenic pathway. In fact, even kinase-dead *BRAF* mutations, which are observed in human cancer, the mutant B-Raf proteins can dimerize with Raf-1, when stimulated by the mutant Ras protein and activate the Raf/MEK/ERK cascade. Clearly for B-Raf-selective inhibitors to be therapeutically useful, prior screening of patients for *RAS* mutations will be mandatory, as well as perhaps additional screening during treatment. Otherwise resistance may develop and lead to further stimulation of the Raf/MEK/ERK cascade.

## MEK INHIBITORS

Specific inhibitors of MEK have been developed (*e.g.,* PD98059 (Pfizer), U0126 (DuPont), PD184352 [CI-1040] (Pfizer), PD0325901 (Pfizer), Selumetinib (*a.k.a*., ARRY-142886, AZD6244) (Astra-Zeneca), and RDEA119 (Ardea Biosciences) (See Table 1) [3, 8-9, 13-30]. MEK inhibitors differ from most other kinase inhibitors as they do not compete with ATP binding (non-ATP competitive), which confers a high specificity [17]. Most MEK inhibitors are specific and do not inhibit many different protein kinases [18] although as will be discussed below, certain MEK inhibitors are more specific than others. The crystal structures of MEK1 and MEK2 have been solved as ternary complexes with ATP and PD184352, and have revealed that both MEK1 and MEK2 have unique inhibitor binding sites located on a hydrophobic pocket adjacent to, but not overlapping with, the ATP-binding site [19]. Furthermore, effective targeting of MEK1/MEK2 is highly specific, as ERK1/ERK2 are the only well-described downstream targets. A distinct advantage of inhibiting MEK is that it can be targeted without knowledge of the precise genetic mutation that results in its aberrant activation. This is not true with targeting Raf as certain Raf inhibitors will activate Raf and also certain B-Raf specific inhibitors will not be effective in the presence of Ras mutations as discussed above.

An advantage of targeting MEK is that the Ras/Raf/MEK/ERK pathway is a convergence point where a number of upstream signaling pathways can be blocked with the inhibition of MEK. For example, MEK inhibitors, such as Selumetinib, are also being investigated for the treatment of pancreatic cancers, breast cancers, and other cancers such as hematopoietic malignancies, including multiple myeloma [20-22].

Selumetinib inhibits MEK1 *in vitro* with an IC50 value of 14.1 ± 0.79 nM [23, 24]; it is specific for MEK1 as it did not appear to inhibit any of the approximately 40 other kinases in the panel tested. Selumetinib is not competitive with ATP. Molecular modeling studies indicate that selumetinib binds to an allosteric binding site on MEK1/MEK2. The binding sites on MEK1/MEK2 are relatively unique to these kinases and may explain the high specificity of MEK inhibitors. This binding may lock MEK1/2 in an inactivate conformation that enables binding of ATP and substrate, but prevents the molecular interactions required for catalysis and access to the ERK activation loop. In basic research studies, treatment with the MEK inhibitor results in the detection of activated MEK1/2 when the western blot is probed with an antibody that recognizes active MEK1/2, while downstream ERK1/2 will not appear activated with the activation specific ERK1/2 antibody [24]. Selumetinib inhibited downstream ERK1/ERK2 activation in *in vitro* cell line assays with stimulated and unstimulated cells, and also inhibited activation in tumor-transplant models. Selumetinib did not prevent the activation of the related ERK5 that occurs with some older MEK1 inhibitors, which are not being pursued in clinical trials. Inhibition of ERK1/2 suppresses their ability to phosphorylate and modulate the activity of Raf-1, B-Raf and MEK1 but not MEK2 as MEK2 lacks the ERK1/ERK2 phosphorylation site. In essence, by inhibiting ERK1/2 the negative loop of Raf-1, B-Raf and MEK phosphorylation is suppressed and hence there will be an accumulation of activated Raf-1, B-Raf and MEK [24]. This biochemical feedback loop may provide a rationale for combining Raf and MEK inhibitors in certain therapeutic situations.

In colon, melanoma, pancreatic, liver and some breast cancers, selumetinib inhibited the growth of tumors in tumor xenograft studies performed in mice. The new MEK inhibitors are also at least 10 to 100-fold more effective than earlier MEK inhibitors and hence can be used at lower concentrations [8, 9, 20-24]. Selumetinib also inhibits the growth of human leukemia cells, but does not affect the growth of normal human cells. Selumetinib also suppressed the growth of pancreatic BxPC3 cells, which do not have a known mutation in this pathway, suggesting that this drug may also be useful for treating cancers that lack definable mutations. However, it is likely that BxPC3 cells have some type of upstream gene mutation/amplification or autocrine growth factor loop that results in activation of the Raf/MEK/ERK pathway.

Selumetinib induced G_1_/S cell-cycle arrest in colon and melanoma cancer cell lines and activated caspase-3 and -7 in some cell lines (Malme3M and SKMEL2); however, caspase induction was not observed in other melanoma (SKMEL28) or colon cancer cell lines (HT29), demonstrating that further research needs to be performed with this inhibitor to determine if it normally induces apoptosis and whether the induction of apoptosis can be increased with other inhibitors or chemotherapeutic drugs.

Selumetinib suppressed the tumor growth of pancreatic cells, such as BxPC3, in immunocompromised mice more effectively than conventional chemotherapeutic drugs, such as gemcitabine, which is commonly used to treat pancreatic cancer; however, once treatment with selumetinib was discontinued, the tumors regrew [21]. Most likely MEK inhibitors do not induce apoptosis, but rather, they inhibit proliferation. That is, MEK inhibitors are cytostatic.

An additional MEK inhibitor is PD-0325901 (Pfizer) [27-30], which follows on from the earlier MEK inhibitors PD-98059 and PD-184352, both of which have been extensively examined in preclinical investigations to determine the role of MEK in various biochemical processes. PD-184352 was the first MEK inhibitor to enter clinical trials and it demonstrated inhibition of activated ERK and anti-tumor activity in patients [25,26]; however, subsequent multicenter, phase II studies with patients with diverse solid tumors did not demonstrate encouraging results [27]. This was probably due to low oral bioavailability and high metabolism, which led to plasma drug levels that were inadequate to suppress tumor growth.

The newer PD-0325901 MEK inhibitor is an orally-active, potent, specific, non-ATP competitive inhibitor of MEK. PD-0325901 demonstrated improved pharmacological and pharmaceutical properties compared with PD-184352, including a greater potency for inhibition of MEK, and higher bioavailability and increased metabolic stability. PD-0325901 has a Ki value of 1 nM against MEK1 and MEK2 in *in vitro* kinase assays. PD-0325901 inhibits the growth of cell lines that proliferate in response to elevated signaling of the Raf/MEK/ERK pathways [27]. Clinical trials with PD-0325901 have documented some successes and some adverse side effects [27-29]. Pfizer has suspended it evaluation in clinical trials. This may have resulted in part from the design of the clinical trials as MEK inhibitors may not be appropriate to treat all types of cancer. MEK inhibitors may be appropriate to treat only those cancers that proliferate in response to activation of the Raf/MEK/ERK pathway [30-32]. Furthermore, it may also be important to include a chemotherapeutic drug or radiation treatment to induce death of the cancer cell.

Raf is also a key therapeutic target [31-34], which lies upstream of MEK. Hence, targeting MEK is an approach to target tumors containing activated RAF genes. The BRAF^V600E^ mutation is present in approximately 6 to 8% of human cancers (overall). Interestingly, approximately 5% of lung cancers have mutations at BRAF which are not at V600E [35]. The effects of PD-0325901 were examined in conditional BRAF^V600E^ tumor models where genetically modified mice express normal B-Raf prior to Cre-mediated recombination, after which they express B-Raf^V600E^ at physiological levels [35]. When B-Raf^V600E^ was induced, the mice developed lung tumors which could be inhibited by PD-0325901 (25 mg/kg/day for approximately two weeks, followed by 12.5 mg/kg/day for an additional two weeks). In contrast, mice treated with vehicle alone developed adenomas. This model indicates that in some cases for MEK inhibitors to yield successful outcomes, the therapy needs to include a cytotoxic drug, as the MEK inhibitors are cytostatic and often as soon as the MEK inhibitors are removed, the tumor may re-emerge.

There are few current effective therapies for HCC [36-39]. Hence targeting signaling pathways activated in HCC has been considered an approach to target HCC. Human HCC tumors have higher expression and enhanced activity of MEK1/2 and ERK1/2 compared with adjacent non-neoplastic liver [37]. Over-expression of activated MEK1 in HCC HepG2 cells resulted in enhanced tumor growth *in vivo* [38]. On the other hand, preclinical studies have demonstrated the potential of MEK inhibition to suppress hepatoma cell proliferation and tumorigenicity [9]. Huynh et al. recently reported that treatment of human HCC xenografts with Selumetinib blocked ERK1/2 activation, reduced *in vivo* tumor growth, and induced apoptosis [9]. Moreover, targeting MEK with PD-0325901 had *in vivo* chemopreventive effects on HCC development in an animal model employing TGF-α-transgenic mice in which liver cancers were induced by diethylnitrosamine treatment [39]. Therefore, MEK represents a potential therapeutic target for HCC.

RDEA119 is a more recently described MEK inhibitor developed by Ardea Biosciences [16]. It is a highly selective MEK inhibitor that displays a >100-fold selectivity in kinase inhibition in a panel of 205 kinases. In contrast, in the same kinase specificity analysis, other recently developed MEK inhibitors (*e.g.,* PD0325901) also inhibited the Src and RON kinases.

There are at least two ERK molecules regulated by the Raf/MEK/ERK cascade, ERK1 and ERK2. Little is known about the differential *in vivo* targets of ERK1 and ERK2. The development of specific ERK1 and ERK2 inhibitors is ongoing and may be useful in the treatment of certain diseases such as those leukemias where elevated ERK activation is associated with a poor prognosis (*e.g*., AML, ALL) [40, 41].

Some tumors are resistant to MEK inhibitors because they contain *EGFR, KRAS, PI3KCA* or *PTEN* mutations [6, 42, 43]. Some cells with *EGFR* or *KRAS* mutations are resistant to MEK inhibitors since they can also activate the Ras/PI3K/Akt/mTOR pathway. These studies, which were performed *in vitro* with cells lines and in vivo using xenografts, also demonstrated that PI3K activation and PTEN inactivation were not always equivalent in terms of inhibitor sensitivity. The authors suggested that a possible reason for this phenomenon could be that PTEN has other functions besides the regulation of Akt (*e.g*., protein phosphatase activity). Furthermore these studies demonstrated that the combination of MEK and PI3K pathway inhibitors could be an effective approach to treat certain cancers that had activation of both pathways.

Only certain types of breast cancer are sensitive to MEK inhibitors [43]. Breast cancers can be classified into three types: luminal breast cancers which are usually estrogen receptor positive and have a relatively good prognosis and response rate to hormonal based therapies, HER2-positive breast cancers which have a poor prognosis if untreated but are initially responsive to the HER2 targeting monoclonal antibody Herceptin, and basal-like breast cancers which have a poor prognosis and lack expression of HER2, estrogen and progesterone receptors (referred to as “triple-negative”). Many basal breast cancers express high levels of EGFR which results in activation of the Ras/Raf/MEK/ERK cascade. Hoeflich and colleagues [43] found that basal cell breast cancers expressed a Ras-like expression profile and tested their hypothesis that these breast cancers could be sensitive to MEK inhibitors, providing that they do not have *PI3KCA* mutations or *PTEN* deletions. In contrast many luminal and HER2-amplified tumors are resistant to MEK inhibitors. They also determined that PTEN loss was a negative predictor factor for response to MEK inhibitors. Furthermore, treatment with MEK inhibitors often led to an increase in activated Akt expression, providing the rationale to examine the consequences of co-addition of MEK and PI3K inhibitors. The authors also determined that co-administration of MEK and PI3K inhibitors enhanced killing of the certain breast cancers. Thus the studies by Wee et al, and Hoeflich et al., have shown the concept that elevated PI3K/Akt/mTOR expression will confer resistance to MEK inhibitors. These studies further illustrate a central concept that we have been discussing in this review which is the critical role of genetics in determining the sensitivity to targeted therapy.

Other studies have also indicated that some tumors with *EGFR* mutations are resistant to MEK inhibitors. Mutations at the *BRAF*, *KRAS*, *EGFR* genes or the chromosomal fusion between anaplastic lymphoma kinase (*ALK*) and *ROS* tyrosine kinases are detected in approximately 50% of NSCLC. NSCLC cells with *BRAF* mutations where shown to be more sensitive to MEK inhibitors than NSCLC with mutations in *EGFR*, *KRAS*, or the chimeric fusion between *ALK* and *ROS* [6]. This was determined by screening a large panel of cell lines (n=87) and tumors (n=916). In this study, cells with mutations at *EGFR* were resistant to MEK inhibitors. This may have resulted from the ability of EGFR to activate the PI3K/PTEN/Akt/mTOR pathway which as discussed below has some crucial overlapping targets as the Raf/MEK/ERK pathway. NSCLC patients with *EGFR* mutations should not be treated with MEK (or BRAF) inhibitors as the respective therapies would be ineffectual.

## PI3K/AKT/MTOR INHIBITORS

Many PI3K inhibitors have been developed [44, 45]. These include: LY-294002 [Lilly], Wortmannin, PX-866 [Oncothyreon], GDC-0941 [Genentech], CAL-101 [Calistoga Pharmaceuticals], XL-147 and XL-765 [Exelixis and Sanofi-Aventis]. Some PDK1 inhibitors have been described but they are not specific for PDK1 including OSU-03012 [Arno Therapeutics] and Celecoxib [Pfizer]. Various Akt inhibitors have been developed [46-48]. These include: A-443654 [Abbott Laboratories], GSK690693 [GlaxoSmithKline], VQD-002 (a.k.a. API-2, VioQuest Pharmaceuticals), KP372-1 [QLT, Inc] and Perifosine [AEterna Zentaris/Keryx Biopharmaceuticals]. Inhibitors of downstream mTOR have been developed [49-53]. These include: rapamycin [Wyeth-Pfizer, Sirolimus] and modified rapamycins (rapalogs) (CCI-779, [Torisel, Temsirolimus, Wyeth-Pfizer], AP-23573 [Ridaforolimus, Ariad-Merck] and RAD001 [Afinitor, Everolimus, Novartis]). Rapamycin and the modified rapalogs are mTORC1 inhibitors. Some dual PI3K/mTOR inhibitors have also been developed [42, 54]. These include: (NVP-BEZ235 [Novartis] and PI-103).

There may be benefits to treating patients with an inhibitor which can target both PI3K and mTOR as opposed to treating patients with two inhibitors, that is one targeting PI3K and one targeting mTOR. Perhaps the most obvious benefit would be lowered toxicities. Treatment with a single drug could have fewer side effects than treatment with two separate drugs. The effects of unwanted Akt activation by mTOR inhibition might be decreased upon treatment with a dual kinase inhibitor. Furthermore, the negative side effects of mTOR inhibition on the activation of the Raf/MEK/ERK pathway might be alleviated with the PI3K inhibitor activity in the dual inhibitor. There remains, however, considerable uncertainty about potential toxicity of compounds that inhibit both PI3K and mTOR enzymes whose activities are fundamental to a broad range of physiological processes.

Some of the PI3K inhibitors such as Wortmannin and LY294002 have been used extensively to investigate the role of PI3K in various biological properties but these compounds are not being clinically explored for multiple reasons, including insolubility in aqueous solutions and high toxicity. The modified wortmannin PX-866 is undergoing clinical trials for advanced metastatic cancer by Oncothyreon. GDC-0941 is in clinical trial for advanced solid cancers by Genentech. XL-147 and XL-765 are in clinical trials for advanced solid tumors by Exelixis and Sanofi-Aventis. CAL-101, a PI3Kδ specific inhibitor, is in clinical trials for hematological malignancies by Calistoga Pharmaceuticals. NVP-BEZ235 is in Phase I/II clinical trials for advanced cancer patients by Novartis.

Triciribine (API-2) inhibits phosphorylation in all three Akt isoforms *in vitro* and the growth of tumor cells overexpressing Akt in mouse xenograft models [45]. The mechanism by which triciribine inhibits Akt activity is unknown. Although no studies have been performed with triciribine in preclinical AML models, the drug has been used in a phase I clinical trial in patients with advanced hematologic malignancies, including refractory/relapsed AML. Results from this trial evaluating triciribine administered on a weekly schedule were encouraging and demonstrated that the drug was well-tolerated, with preliminary evidence of pharmacodynamic activity as measured by decreased levels of activated Akt in primary blast cells [55].

The rapalogs have been extensively examined in clinical trials of various cancers including: breast, prostate, pancreatic, brain, leukemia, lymphoma multiple melanoma, HCC, RCC and non small cell lung carcinomas (NSCLC) [49-53]. The rapalogs Torisel and Afinitor are now approved to treat patients with RCC (see below).

mTOR inhibitors initially demonstrated promise, as PTEN is often deleted in various tumors; however, it has been determined that the mTOR pathway has a complicated feedback loop that actually involves suppression of Akt; hence mTOR inhibitors would potentially activate Akt in some cells [2]. When mTORC1 is suppressed by rapamycin, there is increased mTORC2 activity which is the elusive PDK2 that serves to phosphorylate and activate Akt. mTOR can also be regulated by the Ras/Raf/MEK/ERK pathway and mTOR can activate the Ras/Raf/MEK/ERK pathway. This may be another relevant cross-talk between the Ras/Raf/MEK/ERK and the Ras/PI3K/Akt/mTOR pathways, and might offer a further rationale for treatments combining drugs that inhibit both signaling networks. As mentioned earlier, combination of these novel “dual” inhibitors with either a Raf or MEK inhibitor might lead to more effective suppression of cancer growth.

In addition, it is now emerging that, at least in some cell types, rapamycin does not inhibit 4E-BP1 phosphorylation. Small molecules designed for inhibiting the catalytic site of mTOR have shown promising effects on suppression of signalling downstream of mTOR [56, 57]. The development of mTOR specific kinase ATP-competitive inhibitors is currently under intense investigation.

## TREATMENT OF RENAL CELL CARCINOMA (RCC), MELANOMA AND HEPATOCELLULAR CARCINOMA (HCC) WITH SORAFENIB

Due to the broad specificity of Sorafenib (Nexavar™), this drug has been evaluated for the therapy of diverse cancers, including RCC, melanoma and HCC (due to the involvement of the Raf/MEK/ERK cascade, as well as altered VEGR pathway in these cancers) and gastro-intestinal stromal tumors (GIST) (due to the involvement of c-Kit mutations in this cancer) [58-61]. Sorafenib has been approved for the treatment of kidney cancer, including RCC [59]. BRAF is not mutated in RCC, however, VEGFR-2 may be aberrantly expressed as there is dysregulation of its cognate ligand VEGF which can activate VEGFR2 and the Raf/MEK/ERK cascade. Sorafenib is active as a single agent in this disease, probably due to its ability to suppress the activities of multiple signaling pathways activated in RCC, which are required for growth.

As the *BRAF* gene is mutated in approximately 60 to 70% of melanomas, Sorafenib was tested for its ability to suppress melanoma growth in mouse models [60, 61]. The overwhelming majority of *BRAF* mutations occur at V600E. Sorafenib had only modest activity as a single agent in advanced melanoma and it did not appear to be more effective in the treatment of melanomas that are either WT or mutant at the *BRAF* gene, hence it may be targeting a kinase other than B-Raf in these melanomas (*e.g*., VEGFR). Alternatively, it could be targeting an upstream receptor kinase which signals through the Ras/Raf/MEK/ERK cascade. It is relevant to examine the effects of combining Sorafenib with a MEK inhibitor to treat malignant melanoma and certain other cancers. Sorafenib may target the VEGFR and other membrane receptors expressed on the particular cancer cells, whereas the MEK inhibitor would specifically suppress the Raf/MEK/ERK cascade which is abnormally activated by the *BRAF* oncogene or other mutant upstream signaling molecules. To improve the effectiveness of Sorafenib in the therapy of melanoma, it is being combined with standard chemotherapeutic drugs (see below).

Sorafenib, unlike more novel kinase inhibitors that target the mutant versus WT kinase, binds both the WT and mutant V600E B-Raf proteins and retarded the growth of melanoma xenografts in mice [33, 60, 61]. Other more recently developed Raf kinase inhibitors may show higher selectivity toward the mutant as opposed to WT Raf proteins [10, 11].

## TREATMENT OF MELANOMAS, PANCREATIC, COLON, LUNG, BREAST AND HCC WITH SELUMETINIB

Selumetinib is an orally-active MEK1 inhibitor that has undergone phase II clinical trials. It is one of the first MEK1 inhibitors to be evaluated in randomized phase II trials [3, 13, 20-22, 27]. Selumetinib has demonstrated significant tumor suppressive activity in preclinical models of cancer, including melanoma, pancreatic, colon, lung, liver and breast cancer. The effects of Selumetinib are enhanced significantly if the tumor has a mutation that activates the Raf/MEK/ERK signaling pathway. Selumetinib shows great promise in the treatment of pancreatic cancers, which often have mutations in Ras that can lead to downstream Raf/MEK/ERK pathway activation. Due to the frequent detection of pancreatic cancer at advanced stages, it may be necessary to combine signal transduction inhibitor therapy with conventional chemotherapy after surgical removal of the pancreatic cancer if possible.

Selumetinib has undergone several phase I and II clinical trials. A phase I clinical trial to assess the safety, tolerability and pharmacokinetics of selumetinib in patients with various solid malignancies was performed. Phase II clinical trials have compared: (i) the efficacy of selumetinib versus temozolomide in patients with unresectable stage 3 or 4 malignant melanomas, (ii) the efficacy and safety of selumetinib versus capecitabine in patients with advanced or metastatic pancreatic cancer who have failed to respond to gemcitabine therapy, (iii) the efficacy and safety of selumetinib compared with pemetrexed in patients with NSCLC who have previously failed to respond to one or two prior chemotherapy regimens, and (iv) the efficacy and safety of selumetinib versus capectiabine in patients with colorectal cancer who have failed to respond to one or two prior chemotherapy regimens [62]. Initial results from clinical trials have not yielded overwhelming support for the use of MEK inhibitors (see below) as a single therapeutic agent in cancer patients who are not pre-screened for pre-existing activation of the Raf/MEK/ERK pathway [27, 28]. The proper pre-identification of cancer patients who display activation of the Raf/MEK/ERK pathway may be necessary for prescribing MEK inhibitors as part of their therapy, as we have stated previously that MEK inhibitors are cytostatic and not cytotoxic.

## TREATMENT OF RCC AND HCC WITH MTOR INHIBITORS

The modified rapamycins have been approved by the FDA to treat RCC that have been shown to be refractory to other therapies including sunitinib (Sutent) [63]. Recent studies have demonstrated that mTOR inhibition has remarkable activity against a wide range of human cancers in vitro and human tumor xenograft models. The mTOR pathway is known to be up-regulated in a subset of HCC patients [64]. In this study 15% of HCC displayed overexpression of phospho-mTOR, whereas 45% of HCC had increased expression of p70^S6K^, which correlated with tumor nuclear grade. Evidence from in vitro experiments as well as from preclinical in vivo data indicated that mTOR inhibition by rapamycin and its analogues everolimus (RAD001) significantly reduced the growth of HCC cells and improved survival primarily via antiangiogenic effects [64-67]. A pilot study conducted in 21 patients with advanced HCC indicated that sirolimus (rapamycin) was a promising drug for the treatment of HCC, and currently, a phase I/II trial evaluating the rapamycin analog RAD001 for advanced HCC is recruiting patients (http://clinicaltrials.gov/ct2/show/NCT00390195).

A topic of considerable current interest concerns the signal transduction pathways and the molecular mechanisms linked to chemoresistance of tumor cells to conventional anticancer drugs. In this context, combination of rapamycin with the conventional cytostatic drugs doxorubicin and vinblastine enhances the antineoplastic activity of the respective monotherapeutic HCC treatment with either doxorubicin or vinblastine alone [68, 69]. Taken together, the *in vitro* and preclinical *in vivo* data as well as the clinical trials conducted so far demonstrate that mTOR inhibitors are promising agents for HCC treatment, particularly in combination with conventional chemotherapeutic drug therapy.

## INCREASING THE EFFECTIVENESS OF TARGETING THE RAF/MEK/ERK AND PI3K/PTEN/AKT/MTOR PATHWAYS BY SIMULTANEOUS TREATMENT WITH TWO PATHWAY INHIBITORS

The obvious goal of current inhibitor development is to improve the effectiveness of treatment of cancer patients with small molecule signal transduction inhibitors. This has proven to be difficult for multiple reasons: first, as previously discussed, there tends to be a distinct genetic susceptibility for the success of a signal transduction inhibitor in suppressing growth, second, many of the small molecule signal transduction inhibitors are cytostatic as opposed to being cytotoxic and therefore will need to be combined with a therapeutic modality that induces cell death and will be discussed below and third, more than one signal transduction pathway may be activated in the cancer cells, which will be discussed in detail below.

**Figure 3:**
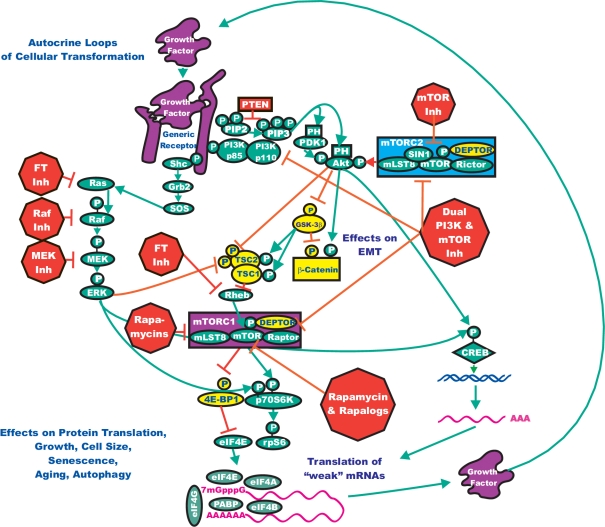
Conceptual Overview of Targeting the Ras/Raf/MEK/ERK and Ras/PI3K/PTEN/Akt/mTOR Pathways to Suppress Malignant Growth The Ras/Raf/MEK/ERK and Ras/PI3K/PTEN/Akt/mTOR pathways can interact at many different levels. In this diagram, we have focused on how they interact to regulate mTOR, p70S6K and protein synthesis and autophagy. Targeting both of these pathways may be an effective means to regulate cell growth. Signaling molecules promoting phosphorylation events are indicated in green. Stimulatory signaling events are indicted in green lines with a green arrow before the target of the phosphorylation. Small molecule inhibitors are indicated in red. Inhibitory phosphorylation events are indicated in red lines with a block on the end before the target of the inhibition. More tentative inhibitory phosphorylation events are indicated in dotted red lines with a block on the end before the target of the inhibition. Inhibitory signaling or proapoptotic molecules or inactivated molecules are indicated in yellow. A growth factor and a growth factor receptor are indicated in purple. Active transcription factors are indicated in purple diamonds. Inactivated transcription factors are indicated in yellow diamonds.

Previously, we have predominantly discussed studies that employed a single Raf or MEK inhibitor, sometimes in combination with a chemotherapeutic drug. In the following section, we discuss the potential of combining inhibitors that target two pathways to more effectively limit cancer growth. In addition to the *BRAF* mutations present in melanomas that we have previously discussed, the *PTEN* phosphatase tumor suppressor gene is also deleted in approximately 45% of melanomas and the downstream *AKT* gene is amplified in approximately 45%. Both of these mutations result in increased expression/activity of Akt which is often associated with a poor prognosis in human cancer. Increased Akt expression will lead to mTOR activation and increased efficiency of protein translation. The targeting of mTOR has been examined in melanoma therapy as well as in the treatment options for many diverse cancers. Administration of mTOR inhibitors to melanoma patients as monotherapy resulted in 1 partial remission out of 33 patients [70]. Preclinical studies performed in human melanoma cell lines have highlighted that co-targeting of the Raf and PI3K/PTEN/Akt/mTOR pathways with Raf and Akt/mTOR inhibitors resulted in synergistic inhibition [71]. Treatment of inducible murine lung cancers containing *KRAS* and *PIK3CA* mutations with PI3K/mTOR (NVP-BEZ235) and MEK (selumetinib) inhibitors led to an enhanced response [72]. Recent reports have also indicated synergistic responses between sorafenib and mTOR inhibitors in xenografts of a highly metastatic human HCC tumor [73]. An illustration documenting the rationale for the targeting of both pathways is presented in Figure 3.

The combined effects of inhibiting MEK with PD-0329501 and mTOR with rapamycin or its analog AP-23573 (ARIAD Pharmaceuticals/Merck) were examined in human NSCLC cell lines, as well as in animal models of human lung cancer [74]. PD-0325901 and rapamycin demonstrated synergistic inhibition of proliferation and protein translation. Suppression of both MEK and mTOR inhibited ribosomal biogenesis and was associated with a block in the initiation phase of translation. These preclinical results support suppression of both the MEK and mTOR pathways in lung cancer therapy and indicate that both pathways converge to regulate the initiation of protein translation. ERK phosphorylates MAPK signal integrating kinases (Mnk1/2) and p90 ribosomal S6 kinase p90^Rsk^, which regulate the activity of the eukaryotic translation initiation factor eIF4E. The phosphorylation of 4EBP1 is altered in cells with the BRAF mutation. It should also be pointed out that the 4EBP1 is also regulated by Akt, mTOR and p70S6K. This may result in the efficient translation of certain mRNAs in BRAF-mutant cells. This could explain how co-inhibition of MEK and mTOR synergize to inhibit protein translation and growth in certain lung cancer cells.

## ENHANCING EFFECTIVENESS OF RAF/MEK AND PI3K/MTOR INHIBITORS WITH CHEMOTHERAPY

**Figure 4:**
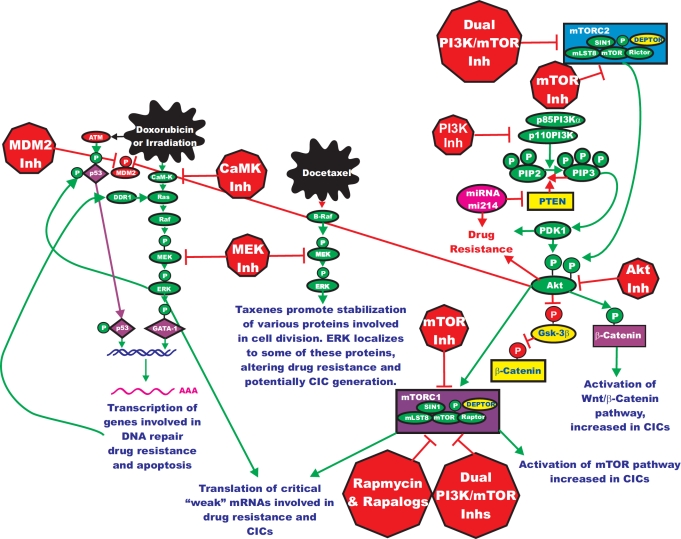
Targeting Ras/Raf/MEK/ERK and Ras/PI3K/PTEN/Akt/mTOR Pathways May Prevent Drug Resistance and Reemergence of Cancer Initiating Cells Chemotherapeutic drugs such as Doxorubicin and Docetaxel may also induce the Raf/MEK/ERK pathway which may contribute to emergence of drug resistant clones. The Raf/MEK/ERK pathway may regulate downstream transcription factors such as GATA-1 which control the transcription of genes such as XRCC1 and ERCC1 which are involved in DNA repair and their aberrant expression may contribute to drug resistance. Treatment of drug resistant cells with MEK inhibitors, or combined treatments consisting of a chemotherapeutic drug and a MEK inhibitor, may be an effective approach to prevent drug resistance. Treatment of certain cancer initiating cells with Akt or mTOR inhibitors may prevent their reemergence. Various components of the Ras/PI3K/PTEN/Akt/mTOR pathway are implicated in drug resistance. Changes in Akt expression may occur to mutations at PI3K or PTEN. Furthermore, altered expression of microRNAs may be involved in decreasing PTEN expression which results in drug resistance. The roles of these various genetic changes in cancer initiating cells are beginning to become apparent. Chemotherapeutic drugs are indicated in irregular black elipses. Treatment of certain cancer initiating cells with Akt or mTOR inhibitors may prevent their reemergence. Signaling molecules promoting phosphorylation events are indicated in green. Stimulatory signaling events are indicted in green lines with a green arrow before the target of the phosphorylation. Small molecule inhibitors are indicated in red. Inhibitory phosphorylation events are indicated in red lines with a block on the end before the target of the inhibition. More tentative inhibitory phosphorylation events are indicated in dotted red lines with a block on the end before the target of the inhibition. Inhibitory signaling or proapoptotic molecules or inactivated molecules are indicated in yellow. A growth factor and a growth factor receptor are indicated in purple. Active transcription factors are indicated in purple diamonds. Inactivated transcription factors are indicated in yellow diamonds.

Classical chemotherapy often remains the most prescribed anti-cancer therapy for many different types of cancer treatment [75]. Drugs such as doxorubicin and taxol are effective in the treatment of many cancers, even though in some cases drug resistance develops after prolonged treatment. Doxorubicin and taxol target cellular events, such as DNA replication and cell division, which are often downstream of the targets of signal transduction pathway inhibitors. Chemotherapeutic drugs can activate the Ras/Raf/MEK/ERK pathway by diverse mechanisms (See Figure 4). Drugs such as doxorubicin can activate p53 which can lead to increased expression of the discoidin domain receptor (DDR), which in turn can result in Raf/MEK/ERK pathway activation. Activated ERK can phosphorylate p53 and regulate its activity. Doxorubicin can also activate the calcium calmodulin dependent kinase (CaM-K) cascade via reactive oxygen species (ROS) [4]. Activation of this cascade can also result in activation of the Raf/MEK/ERK cascade. Activation of this cascade can result in the transcription of genes such as XRCC1 and ERCC1 which are involved in DNA repair and lead to drug resistance [75, 76]. Taxols can also stimulate activation of the Raf/MEK/ERK cascade and lead to their increased association with proteins involved in cell division [77, 78]. Thus, by combining classical chemotherapy with targeted therapy, it may be possible to enhance toxicity, while lowering the prescribed concentrations of classical chemotherapeutics necessary for effective elimination of the tumor [78]. As we have previously discussed, activation of the Raf/MEK/ERK cascade can alter the activity and subcellular localization of many proteins that play critical roles in apoptotic cascades. Also the Raf/MEK/ERK cascade can regulate the transcription of many critical genes involved in cell cycle progression, growth and differentiation.

A phase II trial demonstrated that the combination of sorafenib and doxorubicin improves progression-free and overall survival of patients with advanced HCC [79]. Moreover, a phase II trial is currently recruiting patients to determine the progression-free survival of sorafenib plus tegafur/uracil (UFUR) for the treatment of advanced or metastatic HCC.

As mentioned previously, a side effect of some chemotherapeutic drugs, such as paclitaxel, is the induction of the Raf/MEK/ERK pathways. Activation of this pathway can under certain circumstances promote proliferation and prevent apoptosis. Also the PI3K/PTEN/Akt/mTOR pathway can modulate the Raf/MEK/ERK pathway and altering MEK activity can have opposing effects on different cell types [80, 81]. Combining paclitaxel treatment with PI3K inhibitors enhances apoptosis and inhibits growth of ovarian carcinoma cell lines, and this may have been mediated in part by suppression of inhibitory phosphorylation of Raf by Akt [80]. In addition, the effects of combined treatment with MEK inhibitors and paclitaxel have been examined. The synergistic effects of paclitaxel and MEK inhibitors are complex and have not been fully elucidated, but may be in part mediated by inhibition of Bad phosphorylation at S112 by ERK in UM-SCC-23 squamous carcinoma cell line [82]. This is just one documented interaction that may be suppressed by MEK inhibitors. Obviously many other key phosphorylation events mediated by ERK may be suppressed which play critical roles in cell growth.

The cytotoxic effects of combinations of MEK inhibitors and paclitaxel may be specific for cells of certain origins and may depend on the levels of endogenous activated MEK/ERK present in those cells. In a study with NSCLC cells which constitutively-expressed activated MEK/ERK, no increase in paclitaxel-induced apoptosis was observed when the cells were treated with a MEK inhibitor [81]. In contrast, addition of a dominant negative (DN) MEK gene to these cells potentiated paclitaxel-induced apoptosis.

Cisplatin-induced apoptosis was associated with increased levels of both p53 and the downstream Bax protein in a study with neuroblastoma cells [82]. Activated ERK1/ERK2 levels also increased in these cells upon cisplatin treatment. MEK inhibitors blocked apoptotic cell death, which prevented the cisplatin-induced accumulation of p53 and Bax proteins [82].

It should be noted that the combination of MEK inhibitors and chemotherapeutic drugs may not always result in a positive interaction. In some cases, combination therapy results in an antagonistic response. For example, combining MEK inhibitors with betulinic acid, a drug toxic for melanoma cells, antagonized the normal enhancing effects of betulinic acid on apoptosis *in vitro* [83]. Furthermore, the precise timing of the addition of two agents is important as they may differentially affect cell-cycle progression; therefore, the order of administration may be important for a synergistic response to be obtained and perhaps to prevent an antagonistic response [83].

## ENHANCING EFFECTIVENESS OF RAF/MEK AND PI3K/MTOR INHIBITORS WITH RADIOTHERAPY

Radiotherapy is a common therapeutic approach for treatment of many diverse cancers. A side effect of radiotherapy in some cells is induction of the Ras/Raf/MEK/ERK cascade [4]. Recently various signal transduction inhibitors have been evaluated as radiosensitizers. The effects of pre-treatment of lung, prostate, and pancreatic cancer cells with selumetinib were evaluated *in vitro* using human cell lines and *in vivo* employing xenografts [84]. The MEK inhibitor treatment radiosensitized the various cancer cell lines *in vitro* and *in vivo*. The MEK inhibitor treatment was correlated with decreased Chk1 phosphorylation 1-2 hrs after radiation. The authors noticed the effects of the MEK inhibitor on the G2 checkpoint activation after irradiation, as the MEK inhibitor suppressed G2 checkpoint activation. Since ERK1/ERK2 activity is necessary for carcinoma cells to arrest at the G2 checkpoint, suppression of phosphorylated Chk1 was speculated to lead to the abrogated G2 checkpoint, increased mitotic catastrophe and impaired activation of cell cycle checkpoints. Mitotic catastrophe was increased in cells receiving both the MEK inhibitor and radiation when compared to the solo-treated cells. It was also postulated in this study that the MEK inhibitor suppressed the autocrine cascade in DU145 prostate cancer cells that normally resulted from EGF secretion and EGFR activation. Suppression of this autocrine cascade by the MEK inhibitor may have served as a radiosensitizer to the radiation therapy. The other two cancer cell lines examined in this study (A549 and MiaPaCa2) had *KRAS* mutations and both were radiosensitized by the MEK inhibitor. Although these studies document the ability of a MEK inhibitor to radiosensitize certain cells, clearly other cancer cell lines without activating mutations in the Ras/Raf/MEK/ERK pathway or autocrine growth stimulation should be examined for radiosensitization by the MEK inhibitor as the *KRAS* mutation may also activate the PI3K pathway which could lead to therapy resistance.

PI3K/Akt/mTOR inhibitors will sensitize the tumor vasculature to radiation both *in vitro* in cell lines and *in vivo* in xenogratfs [85, 86]. mTOR and radiation play critical roles in the regulation of autophagy [87, 88]. When mTOR is blocked by rapamycin there is an increase in autophagy. This is important as apoptotic cell death is a minor component to cell death in solid tumors. These studies document the potential beneficial use of combining mTOR inhibitors and radiation to improve the induction of autophagy in the treatment of solid tumors.

Just as new inhibitors are described, cells and tumors resistant to these inhibitors will also be discovered. Resistance to Gleevec (Imatinib) a BCR-ABL inhibitor has been well documented and novel inhibitors have been discovered to overcome this resistance [89]. Recently two distinct mechanisms for resistance to Raf inhibitors have been described [90, 91]. In one case, the *BRAF-*mutant melanoma cells that had been maintained in medium containing the B-Raf inhibitor AZ628 shifted their dependence from B-Raf to Raf-1 [91]. In another case, some B-Raf mutant melanoma cells may be intrinsically resistant to B-Raf inhibitors as a result of cyclin D amplification [91]. Some of these “additional” genetic mutations may be preexisting in the tumor cell population and upon culture of the cells or tumor in the presence of the Raf inhibitor; the “mutant-resistant” cells may take over the population.

## KRAS AND PIK3CA MUTATIONS IN THE SAME CELL OR PATIENT CAN RESULT IN CONFERRING RESISTANCE TO RAPAMYCIN

Cancers containing PIK3CA mutations are often sensitive to the mTOR inhibitor rapamycin and the modified rapamycins (Rapalogs). However, PIK3CA-mutant cells that also have mutations at KRAS are resistant to Rapalogs [92, 93]. This maybe due to complicated feedback loops between the Ras/Raf/MEK/ERK and PI3K/PTEN/Akt/mTOR pathways wherein either mTORC1 inhibition leads to ERK1/2 activation by a p70S6K/PI3K/Ras dependent pathway or by the KRAS mutants activating p90^Rsk-1^ which serves to activate eIF4B and rpS6 thereby bypassing mTOR-dependent activation.

## IDENTIFICATION OF NOVEL SITES IN THE PIK3CA GENE WHICH CONFER RESISTANCE TO PI3K INHIBITORS

A group of highly-gifted graduate students and their colleagues developed an innovative approach to identify residues in *PIK3CA* that will result in resistance or increased sensitivity to PI3K inhibitors [94]. Frequently mutations in kinases which confer resistance to inhibitors occur in the gatekeeper residues that block drug binding. In an insightful study performed by Zunder and colleagues, they took advantage of the fact that yeast do not contain or express *PIK3CA* and that the product of *PIK3CA* (PI3K) is normally toxic to yeast [94]. Therefore introduction of membrane-localized *PIK3CA* into yeast resulted in yeast toxicity, however, when they treated the transfected yeast with a PI3K inhibitor, the yeast survived. They found that certain mutations in *PIK3CA* would confer resistance to the PI3K inhibitors, preventing growth, in transfected yeast at drug concentrations which would allow normal membrane-localized *PIK3CA-*transfected yeast to grow. Unlike with BCR-ABL inhibitor resistant mutations, these *PIK3CA* mutations did not reside in the classic gatekeeper residues. As a biological bonus, they also identified some mutations in *PIK3CA* (L814C) that conferred enhanced sensitivity to PI3K inhibitors. These mutations allowed the growth of the mutant *PIK3CA-*transfected yeast at inhibitor concentrations that would normally suppress the growth of yeast bearing the WT membrane-localized *PIK3CA*. Furthermore, such information is valuable for the design of novel PI3K inhibitors that will be effective in the treatment of cancer patients which become resistant to the first generation of PI3K inhibitors.

## SUMMARY OF RAF/MEK/ERK AND PI3K/PTEN/AKT/MTOR PATHWAYS INHIBITORS EVALUATED IN CANCER THERAPY AND IN CLINICAL TRIALS

In Table 1, a detailed summary of many of the various Raf, MEK, PI3K, Akt and mTOR inhibitors which have been evaluated in preclinical and cancer clinical trials is presented [89, 95-178]. Clearly targeting these activities involved in normal and cancerous growth has become an intensely investigate field. Perhaps some of the most recent success has arisen in targeting mTOR. The regulation of mTOR and its subsequent effects on protein translation is critically implicated in many cancers [171-181] and is also involved in cell differentiation [182-185], cancer initiating cells [187-198] and other important cellular processes as will be discussed below.

## NOVEL USES OF RAF/MEK AND PI3K/AKT/MTOR INHIBITORS: TARGETING CANCER INITIATING CELLS (CICS)

An overview of the Raf/MEK/ERK and PI3K/PTEN/Akt/mTOR pathways in some of novel aspects of their usage is presented in Figure 4. Targeting these pathways may be an approach to overcome chemotherapeutic drug resistance. An area of intense research interest in experimental therapeutics is the cancer stem cell, more appropriately referred to as the cancer initiating cell (CIC) [89, 187-243]. CICs often share some properties with drug resistant cells as they both are often resistant to chemotherapeutic and hormonal based therapies. The abilities of the various Raf, MEK and mTOR inhibitors as well as the natural product resveratrol to target and suppress the proliferation of CICs are beginning to be examined [185, 191-195, 212-220]. It is not clear whether Raf or MEK inhibitors will specifically target CICs [193, 194]. CICs have unique properties from the majority of the particular cancer (often called bulk cancer) as they can be both quiescent and also resistant to chemotherapeutic and hormonal based drugs, often due to their increased expression of proteins involved in drug transport as well as PI3K/PTEN/Akt/mTOR pathway [89, 193, 194, 197-200, 224-226]. However, under certain conditions, they resume proliferation and hence should be potentially susceptible to: Raf, MEK, PI3K, Akt, mTOR and other inhibitors Targeting the Raf/MEK/ERK and PI3K/PTEN/mTOR pathways could be very important in terms of CIC elimination.

The “tumor microenvironment” most likely plays critical roles in CIC survival and also reemergence and subsequent metastasis [206-211]. Combinations of cytotoxic chemotherapeutic drugs and inhibitors which target the Raf/MEK/ERK, PI3K/PTEN/mTOR and upstream kinases may be an eventual approach to target the tumor microenviroment, however, specificity of targeting may be a significant problem. The ability to target the tumor microenvironment is a challenging issue.

Recently miRNAs have been shown to regulate many genes involved in drug resistance and likely CIC regulation [200, 223]. miRNAs specific of the 3'UTR of PTEN have been shown to be upregulated in certain ovarian cancer cells and can cause resistance to cisplatin [223]. One can also hypothesize that there may be altered expression of similar or additional miRNAs in CICs which will alter their sensitivities to mTOR and other inhibitors. The p53 pathway and genome stability/instability play key roles in regulating many aspects of cell growth including CICs [225-243]. We know very little about the changes in p53 and genome stability/instability that may occur in the initial CIC to more “malignant” CICs which may be present at later stages of tumor progression. As we learn more regard the effects of p53 and DNA damage responses on CIC and they development, we may be able to more effectively target these biochemical events from happening and inhibit tumor progression.

## TARGETING THE RAF/MEK/ERK AND PI3K/PTEN/AKT/MTOR PATHWAYS TO SUPPRESS CELLULAR SENESCENCE/QUIESENCE

The Raf/MEK/ERK and PI3K/PTEN/Akt/mTOR pathways also play critical roles in the regulation of cellular senescence and quiescence [227-242]. Escape from drug-induced senescence has also been associated with drug resistance and CICs [227]. Often an additional key molecule implicated in: DNA damage responses, cellular senescence and drug resistance is p53, whose activity can be regulated by both the Raf/MEK/ERK and PI3K/PTEN/Akt/mTOR pathways. These pathways exert their effects on p53 itself (post-translation modification by ERK and many other kinases as well as on the p53 inhibitor MDM-2 [231-236, 239-242]. mTOR can modulate the ability of p53 to promote senescence or quiescence [239, 240]. Genomic instability and epigenomic regulation can also have key effects on CIC generation and cellular senescence/quiescence [233-240] and can be regulated in part by mTOR [239-242].

## TARGETING THE RAF/MEK/ERK AND PI3K/PTEN/AKT/MTOR PATHWAYS TO SUPPRESS CELLULAR AND ORGANISMAL AGING

While we have discussed the roles of these pathways in cancer in significant detail, an additional important aspect of targeting the Raf/MEK/ERK and PI3K/PTEN/Akt/mTOR pathways is to halt cellular aging, which in the end, kills all those who either do not have or have been fortunate to survive cancer and other diseases. There is an emerging scientific field which documents that slowing the growth process and stimulating metabolism will slow aging and perhaps dementia as well [243-273]. Indeed, caloric restriction may be critically important in suppression of aging as well as cancer [263-273]. Many studies have indicated that inhibiting the PI3K/PTEN/Akt/mTOR and Raf/MEK/ERK pathways will influence aging [251, 254, 255, 256]. These experiments have led to innovative hypothesis that cellular senescence results from the hyper-activation of proliferative pathways and that drugs (*e.g.,* Metformin) and signal transduction inhibitors (*e.g.,* Raf, MEK, PI3K, mTOR inhibitors) can inhibit cellular proliferation and cellular aging [251, 254, 255, 256, 271]. Similar effects on the prevention of cellular senescence were observed with Resveratrol, the active component contained in the skins of red grapes which was shown to also inhibit mTOR and p70S6K cellular senescence [193, 194, 252, 256, 257, 259]. Additional studies have shown that the commonly-prescribed diabetes drug Metformin will also inhibit mTOR and prevent cellular aging [246, 247, 266, 270, 271]. Since both the Ras/Raf/MEK/ERK and Ras/PI3K/PTEN/Akt/mTOR pathways interact to regulate the activity of mTOR and downstream components of this pathway are critical for both mRNA stability and protein translation of genes involved in critical growth and survival, it is believed that by inhibiting some of these key pathways, it may be possible to prevent cellular aging.

## CONCLUSIONS

Various pharmaceutical companies have developed inhibitors to the Ras/Raf/MEK/ERK pathway. Initially MEK inhibitors were shown to have the most specificity. However, these inhibitors may have limited effectiveness in treating human cancers, unless the particular cancer proliferates directly in response to the Raf/MEK/ERK pathway. Moreover, MEK inhibitors are often cytostatic as opposed to cytotoxic, thus their ability to function as effective anti-cancer agents in a monotherapeutic setting is limited, and they may be more effective when combined with chemo or radiotherapy. Raf inhibitors have also been developed and some are being used to treat various cancer patients (*e.g.,* Sorafenib). This particular Raf inhibitor also inhibits other receptors and kinases which may be required for the growth of the particular cancer. This promiscuous nature of Sorafenib has contributed to the effectiveness of this particular Raf inhibitor for certain cancers. Mutant specific Raf and PI3K inhibitors are also being developed. This is perhaps the most exciting area in terms of inhibitor development as it may result in the effective targeting of the mutant gene promoting the proliferation of the particular tumor. However, problems have been identified with certain B-Raf mutant allele inhibitors as they will also result in Raf-1 activation if Ras is mutated. Combination therapy with either a traditional drug/physical treatment or another inhibitor that targets a specific molecule in a different signal transduction pathway is also a key approach for improving the effectiveness and usefulness of MEK and Raf inhibitors.

Modified rapamycins, Rapalogs are being used to treat various cancer patients, (*e.g.,* patients with RCC and HCC). While Rapalogs are effective and their toxicity profiles are well know, one inherent property is that they are not very cytotoxic when it comes to killing tumor cells. This inherent property of rapamycins, may also contribute to their low toxicity in humans.

Mutations at many of the upstream receptor genes or Ras can result in abnormal Raf/MEK/ERK and PI3K/PTEN/Akt/mTOR pathway activation. Hence targeting these cascade components with small-molecule inhibitors may inhibit cell growth.. The usefulness of these inhibitors may depend on the mechanism of transformation of the particular cancer. If the tumor exhibits a dependency on the Ras/Raf/MEK/ERK pathway, then it may be sensitive to Raf and MEK inhibitors. In contrast, tumors that do not display enhanced expression of the Ras/Raf/MEK/ERK pathway may not be sensitive to either Raf or MEK inhibitors but if the Ras/PI3K/Akt/mTOR pathway is activated, it may be sensitive to specific inhibitors that target this pathway. Some promising recent observations indicate that certain CICs are sensitive to mTOR inhibitors, documenting their potential use in the elimination of the cells responsible for cancer re-emergence [185, 191]. Some CICs may be sensitive to Resveratrol. Finally, it is likely that many of the inhibitors that we have discussed in this review will be more effective in inhibiting tumor growth in combination with cytotoxic chemotherapeutic drugs or radiation.

Some scientists and clinicians have considered that the simultaneous targeting of Raf and MEK by individual inhibitors may be more effective in cancer therapy than just targeting Raf or MEK by themselves. This is based in part on the fact that there are intricate feed-back loops from ERK which can inhibit Raf and MEK. For example when MEK1 is targeted, ERK1,2 is inhibited and the negative feed-back loop on MEK is broken and activated MEK accumulates. However, if Raf is also inhibited, it may be possible to completely shut down the pathway. This is a rationale for treatment with both MEK and Raf inhibitors. Likewise targeting both PI3K and mTOR may be more effective than targeting either PI3K or mTOR by themselves. If it is a single inhibitor which targets both molecules, such as the new PI3K and mTOR dual inhibitors this becomes a realistic therapeutic option. Finally, an emerging concept is the dual targeting of two different signal transduction pathways, Raf/MEK/ERK and PI3K/PTEN/Akt/mTOR for example. This has been explored in some preclinical models as discussed in the text. The rationale for the targeting of both pathways may be dependent on the presence of mutations in either/or both pathways or in upstream Ras in the particular cancer which can activate both pathways. However, it is not clear, at this point in time, that the targeting of two different kinases in the same pathway or two different kinases in two different pathways with two different inhibitors will be performed clinically in the near future. While it may be scientifically interesting and effective it may be clinically impractical. It might make more clinical sense to target one kinase and also use a chemotherapeutic drug which will kill the cells.

It is not always clear why a particular combination of a signal transduction inhibitor and chemotherapeutic drug works in one tumor type but not at all in a different tumor type. This has also been experience with the development of individual chemotherapeutic drugs, some work in some cells but not others. This may result from many different complex interacting events. Some of these events could include: percentage of cells in different phases of the cell cycle, persistence of CICs and many other factors. Finally, chemotherapeutic drug therapy and other types of therapy (radiotherapy, antibody therapy) may induce certain signalling pathways (*e.g*., the reactive oxygen species generated by chemotherapy and radiotherapy induce the Ras/Raf/MEK/ERK pathway). The induction of these signalling pathways may counteract some of the effects of the signal transduction inhibitors.

Scientists and clinicians often have an intentionally narrow view of a particular topic. For example, cancer researchers predominantly feel that Raf, MEK, PI3K, Akt and mTOR inhibitors will suppress the growth of malignant cancer cells. Yet MEK and mTOR and other inhibitors may also be useful in the treatment of autoimmune and allergic disorder where there is abnormal cellular proliferation. Recently it has been observed that the suppression of the Ras/Raf/MEK/ERK and Ras/PI3K/Akt/mTOR pathways may prevent the induction of cellular senescence and aging. Clearly, these later two clinical topics, immune disorders and aging, greatly enhance the potential clinical uses of these targeted therapeutic drugs.

## References

[1] Steelman LS, Abrams SL, Whelan J, Bertrand FE, Ludwig DE, Basecke J, Libra M, Stivala F, Milella M, Tafuri A, Lunghi P, Bonati A (2008). Contributions of the Raf/MEK/ERK, PI3K/PTEN/Akt/mTOR and JAK/STAT pathways to leukemia. Leukemia.

[2] McCubrey JA, Steelman LS, Abrams SL, Bertrand FE, Ludwig DE, Basecke J, Libra M, Stivala F, Milella M, Tafuri A, Lunghi P, Bonati A (2008). Targeting Survival Cascades Induced by Activation of Raf/Raf/MEK/ERK, PI3K/PTEN/Akt/mTOR and Jak/STAT pathways for effective leukemia therapy. Leukemia.

[3] McCubrey JA, Milella M, Tafuri A, Martelli AM, Lunghi P, Bonati A, Cervello M, Lee JT, Steelman LS (2008). Targeting the Raf/MEK/ERK pathway with small-molecule inhibitors. Current Opinion in Investigational Drugs..

[4] McCubrey JA, Steelman LS, Chappell WH, Abrams SL, Wong EW, Chang F, Lehmann B, Terrian DM, Milella M, Tafuri A, Stivala F, Libra M (2007). Roles of the Raf/MEK/ERK pathway in cell growth, malignant transformation and drug resistance. Biochem Biophys Acta.

[5] Solit DB, Garraway LA, Pratilas CA, Sawai A, Getz G, Basso A, Ye Q, Lobo JM, She Y, Osman I, Golub TR, Sebolt-Leopold J (2006). BRAF mutation predicts sensitivity to MEK inhibition. Nature.

[6] Pratilas CA, Hanrahan AJ, Halilovic E, Persaud Y, Soh J, Chitale D, Shigematsu H, Yamamoto H, Sawai A, Janakiraman M, Taylor BS, Pao W (2008). Genetic predictors of MEK dependence in non-small cell lung cancer. Cancer Res.

[7] Rimassa L, Santoro A (2009). Sorafenib therapy in advanced hepatocellular carcinoma: the SHARP trial. Expert Rev Anticancer Ther.

[8] Huynh H, Soo KC, Chow PK, Tran E (2007). Targeted inhibition of the extracellular signal-regulated kinases kinase pathway with AZD-6244 (ARRY-142886) in the treatment of hepatocellular carcinoma. Mol Cancer Ther.

[9] Casar B, Pinto A, Crespo P (2009). ERK dimmers and scaffold proteins: unexpected partners for a forgotten task. Cell Cycle.

[10] Tsai J, Lee JT, Wang W, Zhang J, Cho H, Mamo S, Bremer R, Gillette S, Kong J, Haass NK, Sproesser K, Li L (2008). Discovery of a selective inhibitor of oncogenic B-Raf kinase with potent antimelanoma activity. Proc Natl Acad Sci USA.

[11] Sala E, Mologni L, Truffa S, Gaetano C, Bollag GE, Gambacorti-Passerini C (2008). BRAF silencing by short hairpin RNA or chemical blockade by PLX-4032 leads to different responses in melanoma and thyroid carcinoma cells. Mol Cancer Res.

[12] Heidorn SJ, Milagre C, Whittaker S, Nourry A, Niculescu-Duvas I, Dhomen N, Hussain J, Reis-Filho JS, Springer CJ, Pritchard C, Marais R (2010). Kinase-dead BRAF and oncogenic RAS cooperate to drive tumor progression through CRAF. Cell.

[13] McCubrey JA, Steelman LS, Abrams SL, Chappell WH, Russo S, Ove R, Milella M, Tafuri A, Lunghi P, Bonati A, Stivala F, Nicoletti F (2010). Emerging MEK Inhibitors. Exp Opin Emerging Drugs.

[14] Abrams SL, Steelman LS, Shelton JG, Wong ET, Chappell WH, Bäsecke J, Stivala F, Donia M, Nicoletti F, Libra M, Martelli AM, McCubrey JA (2010). The Raf/MEK/ERK pathway can govern drug resistance, apoptosis and sensitivity to targeted therapy. Cell Cycle.

[15] Iverson C, Larson G, Lai C, Yeh LT, Dadson C, Weingarten P, Appleby T, Vo T, Maderna A, Vernier JM, Hamatake R, Miner JN (2009). RDEA119/BAY 869766: a potent, selective, allosteric inhibitor of MEK1/2 for the treatment of cancer. Cancer Res..

[16] McCubrey JA, Steelman LS, Kempf CR, Chappell WH, Abrams SL, Stivala F, Malaponte G, Nicoletti F, Libra M, Bäsecke J, Maksimovic-Ivanic D, Mijatovic S (2011). Therapeutic resistance resulting from mutations in Raf/MEK/ERK and PI3K/PTEN/Akt/mTOR signaling pathways. J Cell Physiol.

[17] Delaney AM, Printen JA, Chen H, Fauman EB, Dudley DT (2002). Identification of a novel mitogen-activated protein kinase kinase activation domain recognized by the inhibitor PD 184352. Mol Cell Biol.

[18] Davies SP, Reddy H, Caivano M, Cohen P (2000). Specificity and mechanism of action of some commonly used protein kinase inhibitors. Biochem J.

[19] Ohren JF, Chen H, Pavlovsky A, Whitehead C, Zhang E, Kuffa P, Yan C, McConnell P, Spessard C, Banotai C, Mueller WT, Delaney A (2004). Structures of human MAP kinase kinase 1 (MEK1) and MEK2 describe novel noncompetitive kinase inhibition. Nat Struct Mol Biol.

[20] Tai YT, Fulciniti M, Hideshima T, Song W, Leiba M, Li XF, Rumizen M, Burger P, Morrison A, Podar K, Chauhan D, Tassone P (2007). Targeting MEK induces myeloma-cell cytotoxicity and inhibits osteoclastogenesis. Blood.

[21] Yeh TC, Marsh V, Bernat BA, Ballard J, Colwell H, Evans RJ, Parry J, Smith D, Brandhuber BJ, Gross S, Marlow A, Hurley B (2007). Biological characterization of ARRY-142886 (AZD6244), a potent, highly selective mitogen-activated protein kinase kinase 1/2 inhibitor. Clin Cancer Res.

[22] Wang D, Boerner SA, Winkler JD, LoRusso PM (2007). Clinical experience of MEK inhibitors in cancer therapy. Biochem et Biophysica Acta.

[23] Davies BD, Logie A, McKay JS, Martin P, Steele S, Jenkins R, Cockerill M, Cartlidge S, Smith PD (2007). AZD6244 (ARRY 142886) a potent inhibitor of mitogen-activated protein kinase/extracellular signal-related kinase kinase 1/2 kinases: mechanism of action in vivo, pharmacokinetic/pharmacodynamic relationship and potential for combination in preclinical models. Mol Cancer Ther..

[24] Friday BB, Yu C, Dy GK, Smith PD, Wang L, Thibodeau SN, Adjei AA (2008). BRAF V600E disrupts AZD6244-induced abrogation of negative feedback pathways between extracellular signal-regulated kinase and Raf proteins. Cancer Res.

[25] Allen LF, Sebolt-Leopold J, Meyer MB (2003). CI-1040 (PD184352), a targeted signal transduction inhibitor of MEK (MAPKK). Semin Oncol.

[26] Rinehart J, Adjei AA, LoRusso PM, Waterhouse D, Hecht JR, Natale RB, Hamid O, Varterasian M, Asbury P, Kaldjian EP, Gulyas S, Mitchell DY (2004). Multicenter phase II study of the oral MEK inhibitor, CI-1040, in patients with advanced non-small cell lung, breast, colon, and pancreatic cancer. J Clin Oncol.

[27] Sebolt-Leopold JS (2008). Advances in the development of cancer therapeutics directed against the Ras-mitogen-activated protein kinase pathway. Clin Cancer Res.

[28] Haura EB, Ricart AD, Larson TG, Stella PJ, Bazhenova L, Miller VA, Cohen RB, Eisenberg PD, Selaru P, Wilner KD, Gadgeel SM (2010). A phase II study of PD-0325901, an oral MEK inhibitor, in previously treated patients with advanced non-small cell lung cancer. Clin Cancer Res..

[29] LoRusso PM, Krishnamurthi SS, Rinehart JJ, Nabell LM, Malburg L, Chapman PB, DePrimo SE, Bentivegna S, Wilner KD, Tan W, Ricart AD (2010). Phase I pharmacokinetic and pharmacodynamic study of the oral MAPK/ERK kinase inhibitor PD-0325901 in patients with advanced cancers. Clin Cancer Res..

[30] Mebratu Y, Tesfaigzi Y (2009). How ERK1/2 activation controls cell proliferation and cell death: Is subcellular localization the answer?. Cell Cycle.

[31] McCubrey JA, Steelman LS, Abrams SL, Chappell WH, Russo S, Ove R, Milella M, Tafuri A, Lunghi P, Bonati A, Stivala F, Nicoletti F (2009). Emerging Raf Inhibitors. Exp Opin Emerging Drugs.

[32] Lee JT, Steelman LS, Chappell WH, McCubrey JA (2008). Akt Inactivates ERK causing decreased response to chemotherapeutic drugs in advanced CaP cells. Cell Cycle.

[33] Wilhelm SM, Carter C, Tang LY, Wilkie D, McNabola A, Rong H, Chen C, Zhang X, Vincent P, McHugh M, Cao Y, Shujath J (2004). BAY 43-9006 Exhibits broad spectrum oral antitumor activity and targets the RAF/MEK/ERK pathway and receptor tyrosine kinases involved in tumor progression and angiogenesis. Cancer Research.

[34] Smalley KSM, Flaherty KT (2009). Integrating BRAF/MEK inhibitors into combination therapy for melanoma. British J Can.

[35] Dankort D, Filenova E, Collado M, Serrano M, Jones K, McMahon M (2007). A new mouse model to explore the initiation, progression, and therapy of BRafV600E-induced lung tumors. Genes & Development.

[36] Cusimano A, Azzolina A, Iovanna JL, Bachvarov D, McCubrey JA, Alessandro ND, Monalto G, Cervello M (2010). Novel combination of celecoxib and proteasome inhibitor MG132 in human hepatocellular carcinoma provides synergistic anti tumor effects. Cell Cycle.

[37] Schmidt CM, McKillop IH, Cahill PA, Sitzmann JV (1997). Increased MAPK expression and activity in primary human hepatocellular carcinoma. Biochem Biophys Res Commun.

[38] Wiesenauer CA, Yip-Schneider MT, Wang Y, Schmidt CM (2004). Multiple anticancer effects of blocking MEK-ERK signaling in hepatocellular carcinoma. J Am Coll Surg.

[39] Wentz SC, Wu H, Yip-Schneider MT, Hennig M, Klein PJ, Sebolt-Leopold, Schmidt CM (2008). Targeting MEK is effective chemoprevention of hepatocellular carcinoma in TGF-alpha-transgenic mice. J Gastrointest Surg..

[40] Ricciardi MR, McQueen T, Chism D, Milella M, Estey E, Kaldjian E, Sebolt-Leopold J, Konopleva M, Andreeff M (2005). Quantitative single cell determination of ERK phosphorylation and regulation in relapsed and refractory primary acute myeloid leukemia. Leukemia.

[41] Gregorj C, Ricciardi MR, Petrucci MT, Scerpa MC, De Cave F, Fazi P, Vignetti M, Vitale A, Mancini M, Cimino G, Palmieri S, Di Raimondo F (2007). ERK1/2 phosphorylation is an independent predictor of complete remission in newly diagnosed adult acute lymphoblastic leukemia. Blood.

[42] Wee S, Jagani Z, Xiang KX, Loo A, Dorsch M, Yao YM, Sellers WR, Lengauer C, Stegmeier F (2009). PI3K pathway activation mediates resistance to MEK inhibitors in KRAS mutant cancers. Cancer Res.

[43] Hoeflich KP, O'Brien C, Boyd Z, Cavet G, Guerrero S, Jung K, Januario T, Savage H, Punnoose E, Truong T, Zhou W, Berry L (2009). In vivo antitumor activity of MEK and phosphatidylinositol 3-kinase in basal-like breast cancer models. Clin Cancer Res.

[44] Chiarini F, Fala F, Tazzari PL, Ricci F, Astolfi A, Pession A, Pagliaro P, McCubrey JA, Martelli AM (2009). Dual inhibition of class IA phosphatidylionsitol 3-kinase and mTOR as a new therapeutic option for T-cell acute lymphoblastic leukemia. Cancer Res.

[45] Yang L, Dan HC, Sun M, Liu Q, Sun XM, Feldman RI, Hamilton AD, Polokoff M, Nicosia SV, Herlyn M, Sebti SM, Cheng JQ (2004). Akt/protein kinase B signaling inhibitor-2, a selective small molecule inhibitor of Akt signaling with antitumor activity in cancer cells overexpressing Akt. Cancer Res.

[46] Fala F, Blalock WL, Tazzari P, Cappellini A, Chiarini F, Martinelli G, Tafuri A, McCubrey JA, Cocco L, Martelli AM (2008). Proapoptotic activity and chemosensitizing effect of the novel Akt inhibitor (2S)-1-(1H-Indol-3-yl)-3-[5-(3-methyl-2H-indazol-5-yl)pyridin-3-yl]oxypropan2-amine (A443654) in T acute lymphoblastic leukemia. Molecular Pharmacology.

[47] Mandal M, Younes M, Swan EA, Jasser SA, Doan D, Yigitbasi O, McMurphey A, Ludwick J, El-Naggar AK, Bucana C, Mills GB, Myers JN (2006). The Akt inhibitor KP372-1 inhibits proliferation and induces apoptosis and anoikis in squamous cell carcinoma of the head and neck. Oral Oncol.

[48] Tazzari PL, Tabellini G, Ricci F, Papa V, Bortul R, Chiarini F, Evangelisti C, Martinelli G, Bontadini A, Cocco L, McCubrey JA, Martelli AM (2008). Synergistic proapoptotic activity of recombinant trail plus the akt inhibitor perifosine in acute myelogenous leukemia cells. Cancer Res.

[49] Owonikoko T, Khuri ER, Ramalingam SS (2009). Preoperative therapy for early-stage NSCLC: oppurtunities and challenges. Oncology.

[50] Tamburini J, Green AS, Chapuis N, Bardet V, Lacombe C, Mayeux P, Bouscary D (2009). Targeting translation in acute myeloid leukemia: a new paradigm for therapy?. Cell Cycle.

[51] Donia M, McCubrey JA, Bendtzen K, Nicoletti F (2010). Potential use of rapamycin in HIV infection. Br J Clin Pharmacol..

[52] Fouladi M, Laningham F, Wu J, O'Shaughnessy MA, Molina K, Broniscer A, Spunt SL, Luckett I, Stewart CF, Houghton PJ, Gilbertson RJ, Furman WL (2007). Phase I study of Everolimus in pediatric patients with refractory solid tumors. JCO.

[53] Krymskaya VP, Goncharova EA (2009). PI3K/mTORC1 activation in hamartoma syndromes: therapeutic prospects. Cell Cycle.

[54] Maira SM, Stauffer F, Brueggen J, Furet P, Schnell C, Fritsch C, Brachmann S, Chène P, De Pover A, Schoemaker K, Fabbro D, Gabriel D (2008). Identification and characterization of NVP-BEZ234, a new orally available dual PI3K/mTOR inhibitor with potent in vivo anti tumor activity. Mol Can Ther.

[55] Garrett CR, Coppola D, Wenham RM, Cubitt CL, Neuger AM, Frost TJ, Lush RM, Sullivan DM, Cheng JQ, Sebti SM (2010). Phase I pharmacokinetic and pharmacodynamic study of triciribine phosphate monohydrate, a small-molecule inhibitor of AKT phosphorylation, in adult subjects with solid tumors containing activated AKT. Invest New Drugs..

[56] Feldman ME, Apsel B, Uotila A, Loewith R, Knight ZA, Ruggero D, Shokat KM (2009). Active-site inhibitors of mTOR target rapamycin-resistant outputs of mTORC1 and mTORC2. PLoS Biol.

[57] Thoreen CC, Kang SA, Chang JW, Liu Q, Zhang J, Gao Y, Reichling LJ, Sim T, Sabatini DM, Gray NS (2009). An ATP-competitive mammalian target of rapamycin inhibitor reveals rapamycin-resistant functions of rapamycin inhibitor reveals rapamycin-resistant functions of mTORC1. J Biol Chem.

[58] Llovet JM, Ricci S, Mazzaferro V, Hilgard P, Gane E, Blanc JF, de Oliveira AC, Santoro A, Raoul JL, Forner A, Schwartz M, Porta C (2008). Sorafenib in advanced hepatocellular carcinoma. N Engl J Med.

[59] Escudier B, Eisen T, Stadler WM, Szczylik C, Oudard S, Siebels M, Negrier S, Chevreau C, Solska E, Desai AA, Rolland F, Demkow T (2007). Sorafenib in advanced clear-cell renal-cell carcinoma. N Engl J Med..

[60] Sharma A, Trivedi NR, Zimmerman MA, Tuveson DA, Smith CD, Robertson GP (2005). Mutant V599E B-Raf regulates growth and vascular development of malignant melanoma tumors. Cancer Res.

[61] Sharma A, Tran MA, Liang S, Sharma AK, Amin S, Smith CD, Dong C, Robertson GP (2006). Targeting mitogen-activated protein kinase/extracellular signal-regulated kinase kinase in the mutant (V600E) B-Raf signaling cascade effectively inhibits melanoma lung metastases. Cancer Res.

[62] Tentler JJ, Nallapareddy S, Tan AC, Spreafico A, Pitts TM, Morelli MP, Selby HM, Kachaeva MI, Flanigan SA, Kulikowski GN, Leong S, Arcaroli JJ (2010). Identification of predictive markers of response to the MEK1/2 inhibitor selumetinib (AZD6244) in K-ras-mutated colorectal cancer. Mol Cancer Ther..

[63] Rini BI, Campbell SC, Escudier B (2009). Renal cell carcinoma. Lancet..

[64] Schumacher G, Oidtmann M, Rosewicz S, Langrehr J, Jonas S, Mueller AR (2002). Sirolimus inhibits growth of human hepatoma cells in contrast to tacrolimus, which promotes cell growth. Transplant Proc.

[65] Rizell M, Lindner P (2005). Inhibition of mTOR suppresses experimental liver tumours. Anticancer Res.

[66] Semela D, Piguet AC, Kolev M, Schmitter K, Hlushchuk R, Djonov V, Stoupis C, Dufour JF (2007). Vascular remodeling and antitumoral effects of mTOR inhibition in a rat model of hepatocellular carcinoma. J Hepatol.

[67] Rizell M, Andersson M, Cahlin C, Hafström L, Olausson M, Lindnér P (2008). Effects of the mTOR inhibitor sirolimus in patients with hepatocellular and cholangiocellular cancer. Int J Clin Oncol.

[68] Sieghart W, Fuereder T, Schmid K, Cejka D, Werzowa J, Wrba F, Wang X, Gruber D, Rasoul-Rockenschaub S, Peck-Radosavljevic M, Wacheck V (2007). Mammalian target of rapamycin pathway activity in hepatocellular carcinomas of patients undergoing liver transplantation. Transplantation.

[69] Ribatti D, Nico B, Mangieri D, Longo V, Sansonno D, Vacca A, Dammacco F (2007). In vivo inhibition of human hepatocellular carcinoma related angiogenesis by vinblastine and rapamycin. Histol Histopathol.

[70] Margolin K, Longmate J, Baratta T, Synold T, Christensen S, Weber J, Gajewski T, Quirt I, Doroshow JH (2005). CCI-779 in metastatic melanoma: a phase II trial of the California cancer consortium. Cancer.

[71] Molhoek KR, Brautigan DL, Slingluff CL Jr (2003). Synergistic inhibition of human melanoma proliferation by combination treatment with B-Raf inhibitor BAY43-9006 and mTOR inhibitor rapamycin. J Transl Med.

[72] Engleman JA, Chen L, Tan X, Crosby K, Guimaraes AR, Upadhyay R, Maira M, McNamara K, Perera SA, Song Y, Chirieac LR, Kaur R (2008). Effective use of PI3K and MEK inhibitors to treat mutant Kras G12D and PIK3CA H104R murine lung cancers. Nat Med.

[73] Wang Z, Zhou J, Fan J, Qiu SJ, Yu Y, Huang XW, Tang ZY (2008). Effects of rapamycin alone and in combination with sorafenib in an orthotopic model of human hepatocellular carcinoma. Clin Cancer Res.

[74] Legrier ME, Yang CP, Yan HG, Lopez-Barcons L, Keller SM, Perez-Soler R, Horwitz SB, McDaid HM (2007). Targeting protein translation in human non-small cell lung cancer via combined MEK and mammalian target of rapamycin suppression. Cancer Res.

[75] Flaherty KT (2006). Chemotherapy and targeted therapy combinations in advanced melanoma. Clin Cancer Res.

[76] Andrieux LO, Fautrei A, Bessard A, Guillouzo A, Baffet G, Langouët S (2007). GATA-1 is essential in EGF-meditated induction of nucleotide excision repair activity and ERCCI expression through ERK2 in human hepatoma cells. Cancer Res.

[77] McDaid HM, Lopez-Barcons L, Grossman A, Lia M, Keller S, Pérez-Soler R, Horwitz SB (2005). Enhancement of the therapeutic efficacy of taxol by the mitogen-activated protein kinase kinase inhibitor CI-1040 in nude mice bearing human heterotransplants. Cancer Res.

[78] Haass NK, Sproesser K, Nguyen TK, Contractor R, Medina CA, Nathanson KL, Herlyn M, Smalley KS (2008). The mitogen-activated protein/extracellular signal-regulated kinase kinase inhibitor AZD6244 (ARRY 142886) induces growth arrest in melanoma cells and tumor regression when combined with docetaxel. Clin Cancer Res.

[79] Abou-Alfa GK, Johnson P, Knox JJ, Capanu M, Davidenko I, Lacava J, Leung T, Gansukh B, Saltz LB (2010). Doxorubicin plus sorafenib vs doxorubicin alone in patients with advanced hepatocellular carcinoma: a randomized trial. JAMA..

[80] Mabuchi S, Ohmichi M, Kimura A, Hisamoto K, Hayakawa J, Nishio Y (2002). Inhibition of phosphorylation of BAD and Raf-1 by Akt sensitizes human ovarian cancer cells to paclitaxel. J Biol Chem.

[81] Brognard J, Dennis PA (2002). Variable apoptotic response of NSCLC cells to inhibition of the MEK/ERK pathway by small molecules or dominant negative mutants. Cell Death Differ.

[82] Aoki K, Ogawa T, Ito Y, Nakashima S (2004). Cisplatin activates survival signals in UM-SCC-23 squamous cell carcinoma and these signal pathways are amplified in cisplatin-resistant squamous cell carcinoma. Oncol Rep.

[83] Rieber M, Rieber MS (2006). Signalling responses linked to betulinic acid-induced apoptosis are antagonized by MEK inhibitor U0126 in adherent or 3D spheroid melanoma irrespective of p53 status. Int J Cancer.

[84] Chung EJ, Brown AP, Asano H, Mandler M, Burgan WE, Carter D, Camphausen K, Citrin D (2009). In vitro and in vivo radiosensitization with AZD6244 (ARRY-142886), an inhibitor of mitogen-activated protein kinase/extracellular signal-regulated kinases 1/2 kinase. Clin Cancer Res.

[85] Edwards E, Geng L, Tan J, Onishko H, Donnelly E, Hallahan DE (2002). Phosphatidylinositol 3-kinase/Akt signaling in the response to vascular endothelium to ionizing radiation. Cancer Res.

[86] Shinohara ET, Cao C, Niermann K, Mu Y, Zeng F, Hallahan DE, Lu B (2005). Enhanced radiation damage of tumor vasculature by mTOR inhibitors. Oncogene.

[87] Paglin S, Lee NY, Nakar C, Fitzgerald M, Plotkin J, Deuel B, Hackett N, McMahill M, Sphicas E, Lampen N, Yahalom J (2005). Rapamycin-sensitive pathway regulates mitochondrial membrane potential, autophagy, and survival in irradiated MCF-7 cells. Cancer Res.

[88] Moretti L, Attia A, Kim KW, Lu B (2007). Crosstalk between Bak/Bax and mTOR signaling regulates radiation induced autophagy. Autophagy.

[89] Misaghian N, Ligresti G, Steelman LS, Bertrand FE, Bäsecke J, Libra M, Nicoletti F, Stivala F, Milella M, Tafuri A, Cervello M, Martelli AM (2009). Targeting the leukemic stem cell—the holy grail of leukemia therapy. Leukemia.

[90] Montagut C, Sharma SV, Shioda T, McDermott U, Ulman M, Ulkus LE, Dias-Santagata D, Stubbs H, Lee DY, Singh A, Drew L, Haber DA (2008). Elevated CRAF as a potential mechanism of acquired resistance to BRAF inhibition in melanoma. Cancer Res.

[91] Smalley KS, Lioni M, Dalla Palma M, Xiao M, Desai B, Egyhazi S, Hansson J, Wu H, King AJ, Van Belle P, Elder DE, Flaherty KT (2008). Increased cyclin D1 expression can mediate BRAF inhibitor resistance in BRAF V600E-mutated melanomas. Mol Cancer Ther.

[92] Di Nicolantonio F, Arena S, Tabernero J, Grosso S, Molinari F, Macarulla T, Russo M, Cancelliere C, Zecchin D, Mazzucchelli L, Sasazuki T, Shirasawa S (2010). Deregulation of the PI3K and KRAS signaling pathways in human cancer cells determines their response to everolimus. J Clin Invest.

[93] Mohseni M, Park BH (2010). PIK3CA and KRAS mutations predict for response to everolimus therapy: now that's RAD001. J Clin Invest.

[94] Zunder ER, Knight ZA, Houseman BT, Apsel B, Shokat KM (2008). Discovery of drug-resistant and drug-sensitizing mutations in the oncogenic PI3K isoform p110α. Cancer Cell.

[95] Yang H, Kong W, He L, Zhao JJ, O'Donnell JD, Wang J, Wenham RM, Coppola D, Kruk PA, Nicosia SV, Cheng JQ (2008). Micro RNA expression profiling in human ovarian cancer: miR-274 induces cell survival and cisplatin resistance by targeting PTEN. Cancer Res.

[96] Ouyang B, Knauf JA, Smith EP, Zhang L, Ramsey T, Yusuff N, Batt D, Fagin JA (2006). Inhibitors of Raf kinase activity block growth of thyroid cancer cells with RET/PTC or BRAF mutations in vitro and in vivo. Clin Cancer Res.

[97] Sathornsumetee S, Hjelmeland AB, Keir ST, McLendon RE, Batt D, Ramsey T, Yusuff N, Rasheed BK, Kieran MW, Laforme A, Bigner DD, Friedman HS (2006). AAL881, a novel small molecule inhibitor of RAF and vascular endothelial growth factor receptor activities, blocks the growth of malignant glioma. Cancer Res.

[98] Schwartz GK, Robertson S, Shen A (2009). A phase I study of XL281, a selective oral RAF kinase inhibitor, in patients (Pts) with advanced solid tumors. J Clin Oncol.

[99] King AJ, Patrick DR, Batorsky RS, Ho ML, Do HT, Zhang SY, Kumar R, Rusnak DW, Takle AK, Wilson DM, Hugger E, Wang L (2006). Demonstration of a genetic therapeutic index for tumors expressing oncogenic BRAF by the kinase inhibitor SB-590885. Cancer Res.

[100] Lefloch R, Pouyssegur J, Lenormand P (2009). Total ERK1/2 activity regulates cell proliferation. Cell Cycle.

[101] Wang S, Koromilas AE (2009). Stat1 is an inhibitor of Ras-MAPK signaling and Rho small GTPase expression with implications in the transcriptional signature of Ras transformed cells. Cell Cycle.

[102] Dudley DT, Pang L, Decker SJ, Bridges AJ, Saltiel AR (1995). A synthetic inhibitor of the mitogen-activated protein kinase cascade. Proc Natl Acad Sci USA.

[103] Borysova MK, Cui Y, Snyder M, Guadagon TM (2008). Knockdown of B-Raf impairs spindle formation and the mitotic checkpoint in human somatic cells. Cell Cycle.

[104] Favata MF, Horiuchi KY, Manos EJ, Daulerio AJ, Stradley DA, Feeser WS, Van Dyk DE, Pitts WJ, Earl RA, Hobbs F, Copeland RA, Magolda RL (1998). Identification of a novel inhibitor of mitogen-activated protein kinase kinase. J Biol Chem.

[105] Jilaveanu LB, Zito CR, Aziz SA, Conrad PJ, Schmitz JC, Sznol M, Camp RL, Rimm DL, Kluger HM (2009). C-Raf is associated with disease progression and cell proliferation in a subset of melanomas. Clin Cancer Res..

[106] Takle AK, Bamford MJ, Davies S, Davis RP, Dean DK, Gaiba A, Irving EA, King FD, Naylor A, Parr CA, Ray AM, Reith AD (2008). The identification of potent, selective and CNS penetrant furan-based inhibitors of B-Raf kinase. Bioorg Med Chem Lett.

[107] Vlahos CJ, Matter WF, Hui KY, Brown RF (1994). A specific inhibitor of phosphatidylinositol 3-kinase, 2-(4-morpholinyl)-8-phenyl-4H-1-benzopyran-4-one (LY294002). J Biol Chem.

[108] Sampath D, Cortes J, Estrov Z, Du M, Shi Z, Andreeff M, Gandhi V, Plunkett W (2006). Pharmacodynamics of cytarabine alone and in combination with 7-hydroxystaurosporine (UCN-01) in AML blasts in vitro and during a clinical trial. Blood.

[109] Stengel KR, Dean JL, Seeley SL, Mayhew CN, Knudsen ES (2008). RB status governs differential sensitivity to cytotoxic and molecularly-targeted therapeutic agents. Cell Cycle.

[110] Papa V, Tazzari PL, Chiarini F, Cappellini A, Ricci F, Billi AM, Evangelisti C, Ottaviani E, Martinelli G, Testoni N, McCubrey JA, Martelli AM (2008). Proapoptotic activity and chemosensitizing effect of the novel Akt inhibitor perifosine in acute myelogenous leukemia cells. Leukemia.

[111] Stauffer F, Maira SM, Furet P, García-Echeverría C (2008). Imidazo[4,5-c]quinolines as inhibitors of the PI3K/PKB-pathway. Bioorg Med Chem Lett.

[112] Weisberg E, Banerji L, Wright RD, Barrett R, Ray A, Moreno D, Catley L, Jiang J, Hall-Meyers E, Sauveur-Michel M, Stone R, Galinsky I (2008). Potentiation of antileukemic therapies by the dual PI3K/PDK-1 inhibitor, BAG956: effects on BCR-ABL- and mutant FLT3-expressing cells. Blood.

[113] Rhodes N, Heerding DA, Duckett DR, Eberwein DJ, Knick VB, Lansing TJ, McConnell RT, Gilmer TM, Zhang SY, Robell K, Kahana JA, Geske RS (2008). Characterization of an Akt kinase inhibitor with potent pharmacodynamic and antitumor activity. Cancer Res.

[114] Levy DS, Kahana JA, Kumar R (2009). AKT inhibitor, GSK690693, induces growth inhibition and apoptosis in acute lymphoblastic leukemia cell lines. Blood.

[115] Dudkin L, Dilling MB, Cheshire PJ, Harwood FC, Hollingshead M, Arbuck SG, Travis R, Sausville EA, Houghton PJ (2001). Biochemical correlates of mTOR inhibition by the rapamycin ester CCI-779 and tumor growth inhibition. Clin Cancer Res.

[116] Zeng Z, Sarbassov dos D, Samudio IJ, Yee KW, Munsell MF, Ellen Jackson C, Giles FJ, Sabatini DM, Andreeff M, Konopleva M (2007). Rapamycin derivatives reduce mTORC2 signaling and inhibit AKT activation in AML. Blood.

[117] Carracedo A, Baselga J, Pandolfi PP (2008). Deconstructing feedback-signaling networks to improve anticancer therapy with mTORC1 inhibitors. Cell Cycle.

[118] Yee KW, Zeng Z, Konopleva M, Verstovsek S, Ravandi F, Ferrajoli A, Thomas D, Wierda W, Apostolidou E, Albitar M, O'Brien S, Andreeff M (2006). Phase I/II study of the mammalian target of rapamycin inhibitor everolimus (RAD001) in patients with relapsed or refractory hematologic malignancies. Clin Cancer Res.

[119] Rizzieri DA, Feldman E, Dipersio JF, Gabrail N, Stock W, Strair R, Rivera VM, Albitar M, Bedrosian CL, Giles FJ (2008). A phase 2 clinical trial of deforolimus (AP23573, MK-8669), a novel mammalian target of rapamycin inhibitor, in patients with relapsed or refractory hematologic malignancies. Clin Cancer Res.

[120] Elit L (2006). Drug evaluation: AP-23573--an mTOR inhibitor for the treatment of cancer. IDrugs..

[121] End DW, Smets G, Todd AV, Applegate TL, Fuery CJ, Angibaud P, Venet M, Sanz G, Poignet H, Skrzat S, Devine A, Wouters W (2001). Characterization of the antitumor effects of the selective farnesyl protein transferase inhibitor R115777 in vivo and in vitro. Cancer Res.

[122] Dy GK, Adjei AA (2002). Farnesyltransferase inhibitors in breast cancer therapy. Cancer Invest.

[123] Chao WR, Amin K, Shi Y, Hobbs P, Tanabe M, Tanga M, Jong L, Collins N, Peters R, Laderoute K, Dinh D, Yean D (2010). SR16388: a steroidal antiangiogenic agent with potent inhibitory effect on tumor growth in vivo. Angiogenesis..

[124] Feldman RI, Wu JM, Polokoff MA, Kochanny MJ, Dinter H, Zhu D, Biroc SL, Alicke B, Bryant J, Yuan S, Buckman BO, Lentz D (2005). Novel small molecule inhibitors of 3-phosphoinositide-dependent kinase-1. J Biol Chem.

[125] Peifer C, Alessi DR (2008). Small-molecule inhibitors of PDK1. ChemMedChem..

[126] Bain J, Plater L, Elliott M, Shpiro N, Hastie CJ, McLauchlan H, Klevernic I, Arthur JS, Alessi DR, Cohen P (2007). The selectivity of protein kinase inhibitors: a further update. Biochem J.

[127] Knight ZA, Gonzalez B, Feldman ME, Zunder ER, Goldenberg DD, Williams O, Loewith R, Stokoe D, Balla A, Toth B, Balla T, Weiss WA (2006). A pharmacological map of the PI3-K family defines a role for p110alpha in insulin signaling. Cell.

[128] Fan QW, Knight ZA, Goldenberg DD, Yu W, Mostov KE, Stokoe D, Shokat KM, Weiss WA (2006). A dual PI3 kinase/mTOR inhibitor reveals emergent efficacy in glioma. Cancer Cell.

[129] Prevo R, Deutsch E, Sampson O, Diplexcity J, Cengel K, Harper J, O'Neill P, McKenna WG, Patel S, Bernhard EJ (2008). Class I PI3 kinase inhibition by the pyridinylfuranopyrimidine inhibitor PI-103 enhances tumor radiosensitivity. Cancer Res.

[130] Zou ZQ, Zhang XH, Wang F, Shen QJ, Xu J, Zhang LN, Xing WH, Zhuo RJ, Li D (2009). A novel dual PI3Kalpha/mTOR inhibitor PI-103 with high antitumor activity in non-small cell lung cancer cells. Int J Mol Med.

[131] Yacoub A, Park MA, Hanna D, Hong Y, Mitchell C, Pandya AP, Harada H, Powis G, Chen CS, Koumenis C, Grant S, Dent P (2006). OSU-03012 promotes caspase-independent but PERK-, cathepsin B-, BID-, and AIF-dependent killing of transformed cells. Mol Pharmacol.

[132] Weng SC, Kashida Y, Kulp SK, Wang D, Brueggemeier RW, Shapiro CL, Chen CS (2008). Sensitizing estrogen receptor-negative breast cancer cells to tamoxifen with OSU-03012, a novel celecoxib-derived phosphoinositide-dependent protein kinase-1/Akt signaling inhibitor. Mol Cancer Ther.

[133] Gao M, Yeh PY, Lu YS, Hsu CH, Chen KF, Lee WC, Feng WC, Chen CS, Kuo ML, Cheng AL (2008). OSU-03012, a novel celecoxib derivative, induces reactive oxygen species-related autophagy in hepatocellular carcinoma. Cancer Res.

[134] Ihle NT, Williams R, Chow S, Chew W, Berggren MI, Paine-Murrieta G, Minion DJ, Halter RJ, Wipf P, Abraham R, Kirkpatrick L, Powis G (2004). Molecular pharmacology and antitumor activity of PX-866, a novel inhibitor of phosphoinositide-3-kinase signaling. Mol Cancer Ther.

[135] Howes AL, Chiang GG, Lang ES, Ho CB, Powis G, Vuori K, Abraham RT (2007). The phosphatidylinositol 3-kinase inhibitor, PX-866, is a potent inhibitor of cancer cell motility and growth in three-dimensional cultures. Mol Cancer Ther.

[136] Yu K, Lucas J, Zhu T, Zask A, Gaydos C, Toral-Barza L, Gu J, Li F, Chaudhary I, Cai P, Lotvin J, Petersen R (2005). PWT-458, a novel pegylated-17-hydroxywortmannin, inhibits phosphatidylinositol 3-kinase signaling and suppresses growth of solid tumors. Cancer Biol Ther.

[137] Zhu T, Gu J, Yu K, Lucas J, Cai P, Tsao R, Gong Y, Li F, Chaudhary I, Desai P, Ruppen M, Fawzi M (2006). Pegylated wortmannin and 17-hydroxywortmannin conjugates as phosphoinositide 3-kinase inhibitors active in human tumor xenograft models. J Med Chem.

[138] Walker EH, Pacold ME, Perisic O, Stephens L, Hawkins PT, Wymann MP, Williams RL (2000). Structural determinants of phosphoinositide 3-kinase inhibition by wortmannin, LY294002, quercetin, myricetin, and staurosporine. Mol Cell.

[139] Stein RC (2001). Prospects for phosphoinositide 3-kinase inhibition as a cancer treatment. Endocr Relat Cancer.

[140] Zeng Z, Samudio IJ, Zhang W, Estrov Z, Pelicano H, Harris D, Frolova O, Hail N Jr, Chen W, Kornblau SM, Huang P, Lu Y (2006). Simultaneous inhibition of PDK1/AKT and Fms-like tyrosine kinase 3 signaling by a small-molecule KP372-1 induces mitochondrial dysfunction and apoptosis in acute myelogenous leukemia. Cancer Res.

[141] Koul D, Shen R, Bergh S, Sheng X, Shishodia S, Lafortune TA, Lu Y, de Groot JF, Mills GB, Yung WK (2006). Inhibition of Akt survival pathway by a small-molecule inhibitor in human glioblastoma. Mol Cancer Ther.

[142] Shapiro G, Kwak E, Baselga J (2009). Phase I dose-escalation study of XL147, a PI3K inhibitor administered orally to patients with solid tumors. J Clin Oncol.

[143] Yaguchi S, Fukui Y, Koshimizu I, Yoshimi H, Matsuno T, Gouda H, Hirono S, Yamazaki K, Yamori T (2006). Antitumor activity of ZSTK474, a new phosphatidylinositol 3-kinase inhibitor. J Natl Cancer Inst.

[144] Kong D, Okamura M, Yoshimi H, Yamori T (2009). Antiangiogenic effect of ZSTK474, a novel phosphatidylinositol 3-kinase inhibitor. Eur J Cancer.

[145] Molckovsky A, Siu LL (2008). First-in-class, first-in-human phase I results of targeted agents: Highlights of the 2008 American Society of Clinical Oncology meeting. J Hematol Oncol.

[146] Folkes AJ, Ahmadi K, Alderton WK, Alix S, Baker SJ, Box G, Chuckowree IS, Clarke PA, Depledge P, Eccles SA, Friedman LS, Hayes A (2008). The identification of 2-(1H-indazol-4-yl)-6-(4-methanesulfonyl-piperazin-1-ylmethyl)-4-morpholin-4-yl-thieno[3,2-d]pyrimidine (GDC-0941) as a potent, selective, orally bioavailable inhibitor of class I PI3 kinase for the treatment of cancer. J Med Chem.

[147] Raynaud FI, Eccles SA, Patel S, Alix S, Box G, Chuckowree I, Folkes A, Gowan S, De Haven Brandon A, Di Stefano F, Hayes A, Henley AT (2009). Biological properties of potent inhibitors of class I phosphatidylinositide 3-kinases: from PI-103 through PI-540, PI-620 to the oral agent GDC-0941. Mol Cancer Ther.

[148] Yao E, Zhou W, Lee-Hoeflich ST, Truong T, Haverty PM, Eastham-Anderson J, Lewin-Koh N, Gunter B, Belvin M, Murray LJ, Friedman LS, Sliwkowski MX (2009). Suppression of HER2/HER3-mediated growth of breast cancer cells with combinations of GDC-0941 PI3K inhibitor, trastuzumab, and pertuzumab. Clin Cancer Res.

[149] Serra V, Markman B, Scaltriti M, Eichorn PJ, Valero V, Guzman M, Botero ML, Llonch E, Atzori F, DiCosimo S, Maira M, Garcia-Echeverria C (2008). NVP-BEZ235, a dual PI3K/mTOR inhibitor, prevents PI3K signaling and inhibits the growth of cancer cells with activating PI3K mutations. Cancer Res.

[150] Liu TJ, Koul D, LaFortune T, Tiao N, Shen RJ, Maira SM, Garcia-Echevrria C, Yung WK (2009). NVP-BEZ235, a novel dual phosphatidylinositol 3-kinase/mammalian target of rapamycin inhibitor, elicits multifaceted antitumor activities in human gliomas. Mol Cancer Ther.

[151] Ballou LM, Lin RZ (2008). Rapamycin and mTOR kinase inhibitors. J Chem Biol.

[152] Teachey DT, Grupp SA, Brown VI (2009). Mammalian target of rapamycin inhibitors and their potential role in therapy in leukaemia and other haematological malignancies. Br J Haematol.

[153] Marone R, Erhart D, Mertz AC, Bohnacker T, Schnell C, Cmilianovic V, Stauffer F, Garcia-Echeverria C, Giese B, Maira SM, Wymann MP (2009). Targeting melanoma with dual phosphoinositide 3-kinase/mammalian target of rapamycin inhibitors. Mol Cancer Res.

[154] Pomel V, Klicic J, Covini D, Church DD, Shaw JP, Roulin K, Burgat-Charvillon F, Valognes D, Camps M, Chabert C, Gillieron C, Françon B (2006). Furan-2-ylmethylene thiazolidinediones as novel, potent, and selective inhibitors of phosphoinositide 3-kinase gamma. J Med Chem.

[155] Takle AK, Brown MJ, Davies S, Dean DK, Francis G, Gaiba A, Hird AW, King FD, Lovell PJ, Naylor A, Reith AD, Steadman JG (2006). The identification of potent and selective imidazole-based inhibitors of B-Raf kinase. Bioorg Med Chem Lett.

[156] Edwards LA, Verreault M, Thiessen B, Dragowska WH, Hu Y, Yeung JH, Dedhar S, Bally MB (2006). Combined inhibition of the phosphatidylinositol 3-kinase/Akt and Ras/mitogen-activated protein kinase pathways results in synergistic effects in glioblastoma cells. Mol Cancer Ther.

[157] Hjelmeland AB, Lattimore KP, Fee BE, Shi Q, Wickman S, Keir ST, Hjelmeland MD, Batt D, Bigner DD, Friedman HS, Rich JN (2007). The combination of novel low molecular weight inhibitors of RAF (LBT613) and target of rapamycin (RAD001) decreases glioma proliferation and invasion. Mol Cancer Ther.

[158] Heim M, Sharifi M, Hilger RA, Scheulen ME, Seeber S, Strumberg D (2003). Antitumor effect and potentiation or reduction in cytotoxic drug activity in human colon carcinoma cells by the Raf kinase inhibitor (RKI) BAY 43-9006. Int J Clin Pharmacol Ther.

[159] Wang X, Wang H, Xu L, Rozanski DJ, Sugawara T, Chan PH, Trzaskos JM, Feuerstein GZ (2003). Significant neuroprotection against ischemic brain injury by inhibition of the MEK1 protein kinase in mice: exploration of potential mechanism associated with apoptosis. J Pharmacol Exp Ther.

[160] Sturgeon SA, Jones C, Angus JA, Wright CE (2008). Advantages of a selective beta-isoform phosphoinositide 3-kinase antagonist, an anti-thrombotic agent devoid of other cardiovascular actions in the rat. Eur J Pharmacol.

[161] Kong D, Yamori T (2009). Advances in development of phosphatidylinositol 3-kinase inhibitors. Curr Med Chem.

[162] Arico S, Pattingre S, Bauvy C, Gane P, Barbat A, Codogno P, Ogier-Denis E (2002). Celecoxib induces apoptosis by inhibiting 3-phosphoinositide-dependent protein kinase-1 activity in the human colon cancer HT-29 cell line. J Biol Chem.

[163] Kulp SK, Yang YT, Hung CC, Chen KF, Lai JP, Tseng PH, Fowble JW, Ward PJ, Chen CS (2004). 3-phosphoinositide-dependent protein kinase-1/Akt signaling represents a major cyclooxygenase-2-independent target for celecoxib in prostate cancer cells. Cancer Res.

[164] Shi Y, Liu X, Han EK, Guan R, Shoemaker AR, Oleksijew A, Woods KW, Fisher JP, Klinghofer V, Lasko L, McGonigal T, Li Q (2005). Optimal classes of chemotherapeutic agents sensitized by specific small-molecule inhibitors of akt in vitro and in vivo. Neoplasia.

[165] Mohapatra S, Chu B, Zhao X, Djeu J, Cheng JQ, Pledger WJ (2009). Apoptosis of metastatic prostate cancer cells by a combination of cyclin-dependent kinase and AKT inhibitors. Int J Biochem Cell Biol.

[166] Evangelisti C, Ricci F, Tazzari P, Chiarini F, Battistelli M, Falcieri E, Ognibene A, Pagliaro P, Cocco L, McCubrey JA (2010). Preclinical testing of the Akt inhibitor triciribine in T-cell acute lymphoblastic leukemia. J of Cellular Physiology.

[167] Evangelisti C, Gaboardi GC, Billi AM, Ognibene A, Tazzori PL, McCubrey JA, Martelli AM (2010). Identification of a functional nuclear export sequence in diacyl glycerol kinase delta. Cell Cycle.

[168] Martelli AM, Papa V, Tazzari PL, Evangelesti C, Chiarini C, Grimaldi C, Ricci F, Martinelli G, Ottaviani E, Pagliaro P, Horn S, Basecke J (2010). Erucylphosphohomocholine, the first intravenously applicable alkylphosphocholine, is cytotoxic to acute myelogenous leukemia cells through JNK2- and PP2-dependent mechanisms. Leukemia.

[169] Cappellini A, Chiarini F, Ognibene A, McCubrey JA, Martelli AM (2009). The cyclin-dependent kinase inhibitor Roscovitine and the nucleoside analog sangivamycin induce apoptosis in caspase-3 deficient breast cancer cells independent of caspase mediated P-glycoprotein cleavage: Implications for therapy of drug resistant breast cancers. Cell Cycle.

[170] Chiarini F, Grimaldi C, Ricci F, Tazzari PL, Evangelisti C, Ognibene A, Battistelli M, Falcieri E, Melchionda F, Pession A, Pagliaro P, McCubrey JA (2010). Activity of the novel dual phosphatidylinositol 3-kinasse/mammalian target of rapamycin inhibitor NVP-BEZ235 against T-cell acute lymphoblastic leukemia. Cancer Res.

[171] Evangelisti C, Ricci F, Tazzari P, Tabellini G, Battistelli M, Falcieri E, Chiarini F, Bortul R, Melchionda F, Pagliaro P, Pesson A, McCubrey JA, Martelli AM (2011). Targeted inhibition of mTORC1 and mTORC2 by active-site mTOR inhibitors has cytotoxic effects in T-cell aculte lymphoblastic leukemia. Leukemia.

[172] Martelli AM, Evangelisti C, Chiarini F, McCubrey JA (2010). The phosphatidylinositol 3-kinase/Akt/mTOR signaling network as a therapeutic target in acute myelogenous leukemia patients. Oncotarget.

[173] Buitenhuis M, Coffer PJ (2009). The role of the PI3K-PKB signaling module in regulation of hematopoiesis. Cell Cycle.

[174] Martelli AM, Evangelisti C, Chiarini F, Grimaldi C, McCubrey JA (2010). The emerging role of the phosphatidylinositol 3-kinase/Akt/mammalian target of rapamycin signaling network in cancer stem cell biology. Cancers.

[175] Martelli AM, Evangelisti C, Chiarini F, Grimaldi C, Cappellini A, Ognibene A, McCubrey JA (2010). The emerging role of the phosphatiylinositol 3-kinase/Akt/mammalian target of rapamycin signaling network in normal myelopoiesis and leukemogensis. Biochim Biophys Act.

[176] Shor B, Gibbons JJ, Abraham RT, Yu K (2009). Targeting mTOR globally in cancer: thinking beyond rapamycin. Cell Cycle..

[177] Fujishita T, Aoki M, Taketo MM (2009). The role of mTORC1 pathway in intestinal tumorigenesis. Cell Cycle.

[178] Choo AY, Blenis J (2009). Not all substrates are treated equally: implications for mTOR, rapamycin-resistance and cancer therapy. Cell Cycle.

[179] Gan B, DePinho RA (2009). mTORC1 signaling governs hematopoietic stem cell quiescence. Cell Cycle.

[180] Salmond RJ, Zamoyska R (2010). How does the mammalian target of rapamycin (mTOR) influence CD8 T cell differentiation?. Cell Cycle.

[181] Rao RR, Li Q, Shrikant PA (2010). Fine-tuning CD8(+) T cell functional responses: mTOR acts as a rheostat for regulating CD8(+) T cell proliferation, survival and differentiation?. Cell Cycle.

[182] Renner AG, Creancier L, Dos Santos C, Fialin C, Recher C, Bailly C, Kruczynski A, Payrastre B, Manenti S (2010). A functional link between polo-like kinase 1 and the mammalian target-of-rapamycin pathway?. Cell Cycle.

[183] Tsang CK, Liu H, Zheng XF (2010). mTOR binds to the promoters of RNA polymerase I- and III-transcribed genes. Cell Cycle.

[184] Kim E, Guan KL (2009). RAG GTPases in nutrient-mediated TOR signaling pathway. Cell Cycle.

[185] Yilmaz OH, Valdez R, Theisen BK, Guo W, Ferguson DO, Wu H, Morrison SJ (2006). PTEN-dependence distinguishes haematopoietic stem cells from leukemia-initating cells. Nature.

[186] Blagosklonny MV (2007). Cancer stem cell and cancer stemloids: from biology to therapy. Cancer Biology & Therapy.

[187] Ligresti G, Militello L, Steelman LS, Cavallaro A, Basile F, Nicoletti F, Stivala F, McCubrey JA, Libra M (2009). PIK3CA mutations in human solid tumors. Cell Cycle.

[188] Miyamoto K, Araki KY, Naka K, Arai F, Takubo K, Yamazaki S, Matsuoka S, Miyamoto T, Ito K, Ohmura M, Chen C, Hosokawa K (2007). Foxo3a is essential for maintenance of the hematopoietic stem cell pool. Cell Stem Cell.

[189] Ito K, Bernardi R, Morotti A, Matsuoka S, Saglio G, Ikeda Y, Rosenblatt J, Avigan DE, Teruya-Feldstein J, Pandolfi PP (2008). PML targeting eradicates quiescent leukaemia-initiating cells. Nature.

[190] Ito K, Bernardi R, Pandolfi PP (2009). A novel signaling network as a critical rheostat for the biology and maintenance of the normal stem cell and the cancer-initiating cell. Genes Devel.

[191] McCubrey JA, Abrams SL, Stadelman K, Chappell WH, Lahair M, Ferland RA, Steelman LS (2010). Targeting signal transduction pathways to eliminate chemotherapeutic drug resistance and cancer stem cells. Advan in Enzy Regul.

[192] McCubrey JA, Chappell WH, Abrams SL, Franklin RA, Long JM, Sattler JA, Kempf CR, Laidler P, Steelman LS (2011). Targeting the cancer initiating cells: the Achilles' heel of cancer. Adv Enzyme Regul.

[193] Lu KH, Chen YW, Tsai PH, Tsai ML, Lee YY, Chiang CY, Kao CL, Chiou SH, Ku HH, Lin CH, Chen YJ (2009). Evaluation of radiotherapy effect in resveratrol-treated medulloblastoma cancer stem-like cells. Childs Nerv Syst..

[194] Shankar S, Nall D, Tang SN, Meeker D, Passarini J, Sharma J, Srivastava RK (2011). Resveratrol inhibits pancreatic cancer stem cell characteristics in human and kras transgenic mice by inhibiting pluripotency maintaining factors and epithelial-mesenchymal transition. PLoS One..

[195] Castellano G, Torrisi E, Ligresti G, Malaponte G, Militello L, Russo A, McCubrey J, Canevari S, Libra M (2009). The involvement of the transcription factor yin yang 1 in cancer development and progression. Cell Cycle.

[196] Gil D, Ciolczyk-Wierzbicka D, Dulinska-Litewka J, Zwawa K, McCubrey JA, Laidler P (2011). The mechanism of contribution of integrin linked kinase (ILK) to epithelial mesenchymal transition. Adv Enzyme Regul.

[197] Lee JY, Nakada D, Yilmaz OH, Tothova Z, Joseph NM, Lim MS, Gilliland DG, Morrison SJ (2010). mTOR Activation induces tumor suppressors that inhibit leukemogenesis and deplete hematopoietic stem cells after Pten deletion. Cell Stem Cell.

[198] Ma S, Lee TK, Zheng BJ, Chan KW, Guan XY (2008). CD133+ cancer stem cells confer chemoresistance by preferential expression of the Akt/PKB survival pathway. Oncogene.

[199] Chen C, Liu Y, Liu R, Ikenoue T, Guan KL, Liu Y, Zheng P (2008). TSC-mTOR maintains quiescence and function of hematopoietic stem cells by repressing mitochondrial biogenesis and reactive oxygen species. J Exp Med.

[200] Zimmerman EI, Dollins CM, Crawford M, Grant S, Nana-Sinkam SP, Richards KL, Hammond SM, Graves LM (2010). Lyn kinase-dependent regulation of miR181 and myeloid cell leukemia-1 expression: implications for drug resistance in myelogenous leukemia. Mol Pharmacol.

[201] Hambardzumyan D, Becher OJ, Rosenblum MK, Pandolfi PP, Manova-Todorova K, Holland EC (2008). PI3K pathway regulates survival of cancer stem cells residing in the perivascular niche following radiation in medulloblastoma in vivo. Genes Dev.

[202] Charles N, Holland EC (2010). The perivascular niche microenvironment in brain tumor progression. Cell Cycle.

[203] Heuser M, Humphries RK (2010). Biologic and experimental variation of measured cancer stem cells. Cell Cycle.

[204] Liu Y, Dean DC (2010). Tumor initiation via loss of cell contact inhibition versus Ras mutation: do all roads lead to EMT?. Cell Cycle.

[205] Hussenet T, Dembele D, Martinet N, Vignaud JM, du Manoir S (2010). An adult tissue-specific stem cell molecular phenotype is activated in epithelial cancer stem cells and correlated to patient outcome. Cell Cycle.

[206] Heddleston JM, Li Z, McLendon RE, Hjelmeland AB, Rich JN (2009). The hypoxic microenvironment maintains glioblastoma stem cells and promotes reprogramming towards a cancer stem cell phenotype. Cell Cycle.

[207] Hazlehurst LA, Argilagos RF, Dalton WS (2007). Beta1 integrin mediated adhesion increases Bim protein degradation and contributes to drug resistance in leukaemia cells. Br J Haematol.

[208] Meads MB, Hazlehurst LA, Dalton WS (2008). The bone marrow microenvironment as a tumor sanctuary and contributor to drug resistance. Clin Cancer Res.

[209] Nair RR, Tolentino J, Hazlehurst LA (2010). The bone marrow microenvironment as a sanctuary for minimal residual disease in CML. Biochem Pharmacol.

[210] Podar K, Chauhan D, Anderson KC (2009). Bone marrow microenvironment and the identification of new targets for myeloma therapy. Leukemia.

[211] Stuart SA, Minami Y, Wang JY (2009). The CML stem cell: evolution of the progenitor. Cell Cycle.

[212] Chapuis N, Tamburini J, Green AS, Willems L, Bardet V, Park S, Lacombe C, Mayeux P, Bouscary D (2010). Perspectives on inhibiting mTOR as a future treatment strategy for hematological malignancies. Leukemia.

[213] Chen Y, Li D, Li S (2009). The Alox5 gene is a novel therapeutic target in cancer stem cells of chronic myeloid leukemia. Cell Cycle.

[214] Steelman LS, Abrams SL, Shelton JG, Chappell WH, Bäsecke J, Stivala F, Donia M, Nicoletti F, Libra M, Martelli AM, McCubrey JA (2010). Dominant roles of the Raf/MEK/ERK pathway in cell cycle progression, prevention of apoptosis and sensitivity to chemotherapeutic drugs. Cell Cycle.

[215] Abrams SL, Steelman LS, Shelton JG, Chappell WH, Bäsecke J, Stivala F, Donia M, Nicoletti F, Libra M, Martelli AM, McCubrey JA (2010). Enhancing therapeutic efficacy by targeting non-oncogene addicted cells with combinations of signal transduction inhibitors and chemotherapy. Cell Cycle.

[216] Zhou J, Wulkuhle J, Zhang H, Gu P, Yang Y, Deng J, Margolick JB, Liotta LA, Petricoin E, Zhang Y (2007). Activation of the PTEN/mTOR/STAT3 pathway in breast cancer stem-like cells is required for viability and maintenance. Proc Natl Acad Sci USA.

[217] Li X, Lewis MT, Huang J, Gutierrez C, Osborne CK, Wu MF, Pavlick A, Zhang X, Chamness GC, Wong H, Rosen J, Chang JC (2008). Intrinsic resistance of tumorigenic breast cancer cells to chemotherapy. J Natl Cancer Inst.

[218] Shafee N, Smith CR, Wei S, Kim Y, Mills GB, Hortobagyi GN, Stanbridge EJ, Lee EY (2008). Cancer stem cells contribute to cisplatin resistance in Brca1/p53-mediated mouse mammary tumors. Cancer Res.

[219] Ginestier C, Wicinski J, Cervera N, Monville F, Finetti P, Bertucci F, Wicha MS, Birnbaum D, Charafe-Jauffret Emmanuelle (2009). Retinoid signaling regulates breast cancer stem cell differentiation. Cell Cycle.

[220] Korkaya H, Paulson A, Charafe-Jauffret E, Ginestier C, Brown M, Dutcher J, Clouthier SG, Wicha MS (2009). Regulation of mammary stem/progenitor cells by PTEN/Akt/b-catenin signaling. PLOS Biology.

[221] Vicente-Duenas C, Perez-Caro M, Abollo-Jimenez F, Cobaleda C, Sanchez-Garcia I (2009). Stem-cell driven cancer: “hands-off” regulation of cancer development. Cell Cycle..

[222] Borovski T, Vermeulen L, Sprick MR, Medema JP (2009). One renegade cancer stem cell?. Cell Cycle.

[223] Peter ME (2009). Let-7 and miR-200 microRNAs: guardians against pluripotency and cancer progression. Cell Cycle.

[224] Mathew G, Timm EA Jr, Sotomayor P, Godoy A, Montecinos VP, Smith GJ, Huss WJ (2009). ABCG2-mediated DyeCycle Violet efflux defined side population in benign and malignant prostate. Cell Cycle.

[225] Alvero AB, Chen R, Fu H-H, Montagna M, Schwartz PE, Rutherford T, Silasi D-A, Steffensen KD, Waldstrom M, Visintin I, Mor G (2009). Molecular phenotyping of human ovarian cancer stem cells unravels the mechanisms for repair and chemoresistance. Cell Cycle.

[226] Bleau A-M, Huse JT, Holland EC (2009). The ABCG2 resistance network of glioblastoma. Cell Cycle.

[227] Sabisz M, Skladonowski A (2009). Cancer stem cells and escape from drug-induced premature senescence in human lung tumor cells: implications for drug resistance and in vitro drug screening models. Cell Cycle.

[228] Garinis GA, Schumacher B (2009). Transcription-blocking DNA damage in aging and longevity. Cell Cycle.

[229] Bazarov AV, Hines WC, Mukhopadhyay R, Beliveau A, Meldoyev S, Zaslavsky Y, Yaswen P (2009). Telomerase activation by c-Myc in human mammary epithelial cells requires additional genomic changes. Cell Cycle.

[230] Mele DA, Bista P, Baez DV, Huber BT (2009). Dipeptidyl-peptidase 2 is an essential survival factor in the regulation of cell quiescence. Cell Cycle.

[231] Perucca P, Cazzalini O, Madine M, Savio M, Laskey RA, Vannini V, Prosperi E, Stivala LA (2009). Loss of p21 CDKN1A impairs entry to quiescence and activates a DNA damage response in normal fibroblasts induced to quiescence. Cell Cycle.

[232] Liu Y, Elf SE, Asai T, Miyata Y, Liu Y, Sashida G, Huang G, Di Giandomenico S, Koff A, Nimer SD (2009). The p53 tumor suppressor protein is a critical regulator of hematopoietic stem cell behavior. Cell Cycle.

[233] Korotchkina LG, Demidenko ZN, Gudkov AV, Blagosklonny MV (2009). Cellular quiescence caused by the Mdm2 inhibitor nutlin-3A. Cell Cycle.

[234] Steelman LS, McCubrey JA (2009). Intriguing novel abilities of Nutlin-3A: induction of cellular quiesence as opposed to cellular senescence-implications for chemotherapy. Cell Cycle.

[235] Jung-Hynes B, Ahmad N (2009). Role of p53 in the anti-proliferative effects of Sirt1 inhibition in prostate cancer cells. Cell Cycle.

[236] Nelson G, Buhmann M, von Zglinicki T (2009). DNA damage foci in mitosis are devoid of p53BP. Cell Cycle.

[237] Li L, Borodyansky L, Yang Y (2009). Genomic instability en route to and from cancer stem cells. Cell Cycle.

[238] Burdach S, Plehm S, Unland R, Dirksen U, Borkhardt A, Staege MS, Muller-Tidow C, Richter GHS (2009). Epigenetic maintenance of stemness and malignancy in peripheral neuroectodermal tumors by EZH2. Cell Cycle.

[239] Korotchkina LG, Leontieva OV, Bukreeva EI, Demidenko ZN, Gudkov AV, Blagosklonny MV (2010). The choice between p53-induced senescence and quiescence is determined in part by the mTOR pathway. Aging.

[240] Demidenko ZN, Korotchkina LG, Gudkov AV, Blagosklonny MV (2010). Paradoxical suppression of cellular senescence by p53. Proc Natl Acad Sci USA.

[241] Pospelova TV, Demidenko ZN, Bukreeva EI, Pospelov VA, Gudkov AV, Blagosklonny MV (2009). Pseudo-DNA damage response in senescent cells. Cell Cycle.

[242] Matthew EM, Hart LS, Astrinidis A, Navaraj A, Dolloff NG, Dicker DT, Henske EP, El-Deiry WS (2009). The p53 target Plk2 interacts with TSC proteins impacting mTOR signaling, tumor growth and chemosensitivity under hypoxic conditions. Cell Cycle.

[243] Blagosklonny MV (2007). Program-like aging and mitochondria: instead of random damage by free radicals. J Cell Biochemistry.

[244] Blagosklonny MV, Campisi J, Sinclair DA (2009). Aging: past, present and future. Aging.

[245] Blagosklonny MV (2007). Paradoxes of aging. Cell Cycle.

[246] Blagosklonny MV, Campisi J (2008). Cancer and aging: more puzzles, more promises. Cell Cycle..

[247] Blagosklonny MV, Hall MN (2009). Growth and aging: a common molecular mechanism. Aging.

[248] Blagosklonny MV (2008). Aging, stem cells, and mammalian target of rapamycin: a prospect of pharmalogical rejuvenation of aging stem cells. Rejuvenation Res.

[249] Blagosklonny MV (2008). Aging: ROS or TOR. Cell Cycle.

[250] Blagosklonny MV (2009). TOR-driven aging: speeding car without brakes. Cell Cycle.

[251] Demidenko ZN, Shtutman M, Blagosklonny MV (2009). Pharmacologic inhibition of MEK and PI-3K converges on the mTOR/S6 pathway to decelerate cellular senescence. Cell Cycle.

[252] Demidenko ZN, Blagosklonny MV (2009). At concentrations that inhibit mTOR, resveratrol suppresses cellular senescence. Cell Cycle.

[253] de Keizer PL, Packer LM, Szypowska AA, Riedl-Polderman PE, van den Broek NJ, de Bruin A, Dansen TB, Marais R, Brenkman AB, Burgering BM (2010). Activation of forkhead box O transcription factors by oncogenic BRAF promotes p21cip1-dependent senescence. Cancer Research.

[254] Demidenko ZN, Blagosklonny MV (2008). Growth stimulation leads to cellular senescence when the cell cycle is blocked. Cell Cycle.

[255] Demidenko ZN, Zubova SG, Bukreeva EI, Pospelov VA, Pospelova TV, Blagosklonny MV (2009). Rapamycin decelerates cellular senescence. Cell Cycle.

[256] Blagosklonny MV (2009). Aging-suppressants: Cellular senescence (hyperactivation) and its pharmacologic decleration. Cell Cycle.

[257] Blagosklonny Mikhail V (2009). Inhibition of S6K by resveratrol: in search of the purpose. Aging.

[258] Heeren G, Rinnerthaler M, Laun P, von Seyerl P, Kossler S, Klinger H, Hager M, Bogengruber E, Jarolim S, Simon-Nobbe B, Schuller C, Carmona-Gutierrez D (2009). The mitochondrial ribosomal protein of the large subunit, Afo1p, determines cellular longevity through mitochondrial back-signaling via TOR1. Aging.

[259] Armour SM, Baur JA, Hsieh SN, Land-Bracha A, Thomas SM, Sinclair DA (2009). Inhibition of mammalian S6 kinase by resveratrol suppresses autophagy. Aging.

[260] Jin J, Wang GL, Salisbury E, Timchenko L, Timchenko NA (2009). GSK3beta-cyclin D3-CUGBP1-eIF2 pathway in aging and in myotonic dystrophy. Cell Cycle.

[261] Sang L, Coller HA (2009). Fear of commitment: Hes1 protects quiescent fibroblasts from irreversible cellular fates. Cell Cycle.

[262] Gems D, Doonan R (2009). Antioxidant defense and aging in C. elegans: is the oxidative damage theory of aging wrong?. Cell Cycle.

[263] Goldberg AA, Richard VR, Kyryakov P, Bourque SD, Beach A, Burstein MT, Glebov A, Koupaki O, Boukh-Viner T, Gregg C, Juneau M, English AM (2010). Chemical genetic screen identifies lithocholic acid as an anti-aging compound that extends yeast chronological life span in a TOR-independent manner, by modulating housekeeping longevity assurance processes. Aging.

[264] Blagosklonny MV (2010). Why men age faster but reproduce longer than women: mTOR and evolutionary perspectives. Aging.

[265] Blagosklonny MV (2010). Why human lifespan is rapidly increasing: solving “longevity riddle” with “revealed-slow-aging” hypothesis. Aging.

[266] Blagosklonny MV (2009). Validation of anti-aging drugs by treating age-related diseases. Aging.

[267] Demidenko ZN, Blagosklonny MV (2009). Quantifying pharmacologic suppression of cellular senescence: prevention of cellular hypertrophy versus preservation of proliferative potential. Aging.

[268] Simpson SJ, Raubenheimer D (2009). Macronutrient balance and lifespan. Aging.

[269] Anisimov VN, Zabezhinski MA, Popovich IG, Piskunova TS, Semenchenko AV, Tyndyk ML, Yurova MN, Antoch MP, Blagosklonny MV (2010). Rapamycin extends maximal lifespan in cancer-prone mice. American J Path.

[270] Blagosklonny MV (2007). An anti-aging drug today: from senescence-promoting genes to anti-aging pill. Drug Discovery Today.

[271] Anisimov VN, Berstein LM, Egormin PA, Piskunova TS, Popovich IG, Zabezhinski MA, Tyndyk ML, Yurova MV, Kovalenko IG, Poroshina TE, Semenchenko AV (2008). Metformin slows down aging and extends life span of female SHR mice. Cell Cycle.

[272] Guenther GG, Edinger AL (2009). A new take on ceramide: starve cells by cutting off the nutrient supply. Cell Cycle.

[273] Blagosklonny MV (2010). Calorie restriction: decelerating mTOR-driven aging from cells to organisms (including humans). Cell Cycle.

